# *De Novo* Analysis of Transcriptome Dynamics in the Migratory Locust during the Development of Phase Traits

**DOI:** 10.1371/journal.pone.0015633

**Published:** 2010-12-30

**Authors:** Shuang Chen, Pengcheng Yang, Feng Jiang, Yuanyuan Wei, Zongyuan Ma, Le Kang

**Affiliations:** State Key Laboratory of Integrated Management of Pest Insects and Rodents, Institute of Zoology, Chinese Academy of Sciences, Beijing, China; University of Nebraska Medical Center, United States of America

## Abstract

Locusts exhibit remarkable density-dependent phenotype (phase) changes from the solitary to the gregarious, making them one of the most destructive agricultural pests. This phenotype polyphenism arises from a single genome and diverse transcriptomes in different conditions. Here we report a *de novo* transcriptome for the migratory locust and a comprehensive, representative core gene set. We carried out assembly of 21.5 Gb Illumina reads, generated 72,977 transcripts with N50 2,275 bp and identified 11,490 locust protein-coding genes. Comparative genomics analysis with eight other sequenced insects was carried out to indentify the genomic divergence between hemimetabolous and holometabolous insects for the first time and 18 genes relevant to development was found. We further utilized the quantitative feature of RNA-seq to measure and compare gene expression among libraries. We first discovered how divergence in gene expression between two phases progresses as locusts develop and identified 242 transcripts as candidates for phase marker genes. Together with the detailed analysis of deep sequencing data of the 4^th^ instar, we discovered a phase-dependent divergence of biological investment in the molecular level. Solitary locusts have higher activity in biosynthetic pathways while gregarious locusts show higher activity in environmental interaction, in which genes and pathways associated with regulation of neurotransmitter activities, such as neurotransmitter receptors, synthetase, transporters, and GPCR signaling pathways, are strongly involved. Our study, as the largest *de novo* transcriptome to date, with optimization of sequencing and assembly strategy, can further facilitate the application of *de novo* transcriptome. The locust transcriptome enriches genetic resources for hemimetabolous insects and our understanding of the origin of insect metamorphosis. Most importantly, we identified genes and pathways that might be involved in locust development and phase change, and may thus benefit pest management.

## Introduction

Locusts are one of the most destructive agricultural pests in the world, causing considerable economic and ecological damage. They are also an important physiological model, as they exhibit responses to environment that manifest themselves as phenotypic changes in behavior, color, metabolism, development, and morphology, known as locust phase polyphenism [1], [2], [3], [4]. Changes in locust population density result in a reversible transformation, in a graded manner, between two extreme phenotypic forms, defined as solitary and gregarious phases. At low population densities, solitary individuals are comparatively inactive and avoid each other. However, when the population density reaches a critical threshold, they aggregate and enter an active, gregarious phase resulting in bands of nymph and migratory swarms of adults, both of which cause millions of dollars worth of agricultural crop losses. This phenotypic plasticity in locusts results from the interplay between the environment and gene expression, including regulation of transcription, translation, and hormonal regulation, etc.

Locust phase polyphenism was first discovered in the migratory locust, *Locusta migratoria* [5]. Since then extensive studies have been carried out on locust morphology, neurophysiology and behavior to explore the locust phase change [6], [7], [8], [9]. Many detailed studies have been made using the desert locust, *Schistocerca gregaria*, and it has been demonstrated that external stimulation including sight, smell and touch by other locusts and endogenous serotonin all trigger gregarious behavior transition in desert locusts [3], [10], [11]. Phase polyphenism is believed to have evolved convergently in locusts [3], but it is not known whether the mechanisms that cause the phase change are similar or not among locust species. Phase polyphenism is also affected by the development process. Generally the phase traits are gradually accumulated during the development process, and many developmental stage-dependent differences in phase traits can be observed [3], but the relationship between phase and development in locusts has not been sufficiently understood.

A major gap in the understanding of locust phase change is its underlying genetic basis [12]. The complete genome of the migratory locust is not yet available because of its unusually large size (∼6500 Mb); it is nearly 2 times larger than the human genome and 36 times larger than the genome of *Drosophila melanogaster* [13]. In the migratory locust, expressed sequence tag (EST) analysis has identified 532 unigenes differentially expressed in the 5^th^ instar [14] and a recent RNA-seq study has shown differences in small RNAs between the two phases [15], indicating important roles played by genetic and epigenetic factors. However, due to the limitations of Sanger sequencing, EST data can not well represent genes other than those most abundantly expressed ones [16], [17]. And also due to the throughput limits, only a small part of the locust gene set has been discovered. Moreover, because of the low redundancy of sequencing reads, EST data are not suitable to quantitatively estimate transcript abundance [16], [18]. Most importantly, the previous locust EST work only studied the 5^th^ instar stage, without investigating development effects on locust phase polyphenism. Given the complexity of locust phase polyphenism, it is necessary to carry out a genome-wide study to systematically gauge molecular differences between the two phases.

The high-throughput nature of next-generation sequencing technologies now make it possible to carry out genome-wide studies of transcriptomes in a rapid way and have been widely used to explore gene structure and expression profiling on model organisms [19], [20], [21], known as RNA-seq. As development of assemblers, the *de novo* transcriptome, using *de novo* assembly of short reads by RNA-seq to carry out gene discovery in organisms without genome reference, newly emerges, but has not been applied to genome-wide large-scale gene discoveries yet. *De novo* transcriptomes to date keep in a small amount of sequencing less than 6 Gb of total sequence data, resulting in contigs or scaffolds with mean length less than 600 bp [22], [23], [24].

Here, in order to achieve a high coverage of the gene content of the migratory locust, we carried out the first *de novo* transcriptome with the aim of developing a comprehensive core gene set of an organism having a huge genome size, and also the largest *de novo* transcriptome (447 million reads, 21.5 Gb), with the best assembly results to date (72,977 sequences having a mean length of 1,170). Our work presents the first example of using *de novo* transcriptome to perform large-scale gene discovery and achieve a high coverage of protein coding genes. To achieve this goal, we optimized our sequencing and assembly strategy. It can considerably improve the assembly results and contribute to the studies of experiment design of the *de novo* transcriptome. Another application of RNA-seq is to measure gene expression profiles. We integrated our gene expression profiling workflow into a pipeline, which is available in our website and could be helpful to other *de novo* transcriptome analysis.

Based on our 12 libraries of gregarious and solitary locusts at various developmental stages, we investigated how phase differences gradually progress as locusts develop and identified genes and pathways that can be used to interrogate the molecular and genetic bases involved in development and phase changes in the migratory locust. Specifically, we discovered a divergence of biological investment of two phases in the molecular level and observed involvement of neurotransmitter activity regulation and GPCR signaling, which we proposed to be possible mechanism of migratory locusts to respond to changes in population density.

## Results

### Assembly strategy and de novo transcriptome assembly

Our sequencing and assembly strategy includes two points: do not pool reads from different samples but carry out assembly separately; guarantee the amount of sequence data for each assembly. We constructed 12 cDNA libraries derived from whole bodies of gregarious and solitary migratory locusts (*Locusta migratoria*) in six different developmental stages: egg, combined 1^st^ and 2^nd^ instar, 3^rd^ instar, 4^th^ instar, 5^th^ instar, and adult, and deeply sequenced two samples: the gregarious and solitary 4^th^ instar locusts (G4 and S4), generating 376 million paired-end (PE) reads (about 19.4 Gb) with read lengths ranging from 45 bp–75 bp. We focused on 4^th^ instar locusts for biological reasons that they display typical gregarious and solitary phase traits and transformation between the two phases is easily reversible in this stage. For other 10 samples we obtained >5 million 35-bp single-end (SE) reads each (Table S1). All reads were submitted to SRA at NCBI under the accession no. SRA020599.22.

We put forward this strategy, as pooling reads from multiple samples will bring in uncertain factors in *de novo* assembly, such as increase of alternative splicing, and thus affect assembly. To evaluate, we performed “separate assembly” by assembling PE reads from G4 and S4 into scaffolds separately, and SE reads from other 10 samples into contigs by using SOAPdenovo [25], [26], and then combined the three parts by using CAP3 [27]; we also performed “pooled assembly” by assembling the pool of all reads using SOAPdenovo, followed by using CAP3 as well. Test results showed that separate assembly had a much better performance than pooled assembly. We first analyzed raw data in the contig and scaffold step in three tested k-mers 19, 21 and 23. Although the input reads number doubled in the pooled assembly using all reads (PAAR) as compared to G4 and S4 (the two main parts in separate assembly), numbers of long contigs (>500 bp, 200∼500 bp) in the output were similar in G4, S4 and PAAR; the number of short contigs (<50 bp) in PAAR was much higher than that in G4 or S4 (Table S2). Similar trend was observed in the scaffold step (Table S3). It seems that in the pooled assembly increase of sequencing depth do not contribute much to sequence elongation, but contribute a lot to generating short ones. After using CAP3 to combine all three parts in the separate assembly and to reduce redundancy in both separate assembly and pooled assembly, we found that the average sequence length, total length and N50 were all higher in the former, and the number of sequences >2 kb was doubled in the former, but those <500 bp was more numerous in the latter (Table 1). Our results demonstrate that the separate assembly strategy can considerably improve *de novo* assembly.

**Table 1:** Statistics of the pooled assembly, the separate assembly and final version of this locust transcriptome

	Scaffolds			After CAP3				Final version
	pooled	separate		pooled	separate			Separate Assembly
	all reads	G4	S4	all reads	G4	S4	all reads	
Length distribution								
100–500	64,140	26,606	50,550	63,592	26,319	50,063	49,996	28,280
500–1k	18,413	8,884	18,048	18,350	8,852	17,988	24,047	18,079
1k–2k	8,240	5,895	8,055	8,210	5,885	8,039	13,660	14,042
> = 2k	5,600	5,479	4,569	5,618	5,486	4,584	10,284	12,576
Total NO.	96,393	46,864	81,222	95,770	46,542	80,674	97,987	72,977
Length statistics (bp)								
Total length (Mb)	58.4	42.1	47.8	58.3	42	47.7	83.1	85.4
Mean	606	898	588	609	903	591	848	1,171
Median	263	403	250	265	407	252	472	647
Min	100	100	100	100	100	100	100	100
Max	25,666	22,875	20,167	25,666	22,875	20,167	22,875	27,681
N50	1,254	2,061	1,138	1,263	2,071	1,143	1,750	2,275

K-mer 23 was used. “Scaffolds” shows results in the scaffold step. CAP3 was used to combine G4, S4 scaffolds and contigs derived from other 10 samples into “all reads” in the separate assembly and to reduce redundancy in both assemblies. The final version was generated by separate assembly and a gap filing step was added before using CAP3.

As a result, we chose separate assembly and k-mer 23, and finally generated 72,977 long transcripts with lengths longer than 100 bp (Table 1). The total length of assembled long transcripts was approximately 85.4 Mb and the N50 size was 2,275 bp, with 26,618 sequences longer than 1 kb.

### Quality evaluation of assembled sequences

Then we used locust EST database [14], 38 full-length cDNAs available in GenBank, and 13 ORFs confirmed experimentally in our laboratory to evaluate the *de novo* assembly results. We first created an EST set (reads covered ESTs) to evaluate assembly efficiency, which contained 11,498 ESTs with >90% coverage by at least 2X Illumina reads, since only regions well covered by reads could be assembled. We found 74.9% of these ESTs were covered by transcriptome sequences with >80% coverage (Figure S1A). Of the cDNAs and ORFs examined, 64.7% were detected in the transcriptome with >75% coverage. For these sequences, the average rate of mismatch in covered regions was 0.00495 (Figure S1B).

We evaluated misassemblies or chimerism by searching against NR database and found 207 out of 23,160 NR annotated sequences matched to more than one protein in NR (identity >0.3, e-value <1e-10, score >200). Misassemblies were also evaluated by PCR cloning experiments, in which assembled sequences were used as templates for PCR primer design. 42 out of 45 were succeeded and the length of validated regions ranged from 565 bp to 2946 bp. Five of them were further validated by full-length cDNA clones using RACE PCR (Figure S1D) and none of them were misassembled.

To know how many new genes or transcripts were discovered in this locust transcriptome, we compared them with unigenes (LocustDB) [28] derived from locust ESTs [14]. 10,590 (14.5%) transcripts in locust transcriptome can be clustered with 9,432 (77.6%) previous unigenes, in the criteria of identity >0.96, overlap >50% in reciprocally searches using BLAT. 62,387 transcripts in this transcriptome were first discovered. Using fruit fly genes as an indicator, 5,861 fly genes (flybase release 5.8) were hit (e-value <1e-5) by unigenes, 4,473 more fly genes were hit in the transcriptome, and reciprocal best hits increased from 2,234 by unigenes to 5,478 by transcriptome.

### Locust gene content

**Figure 1:**
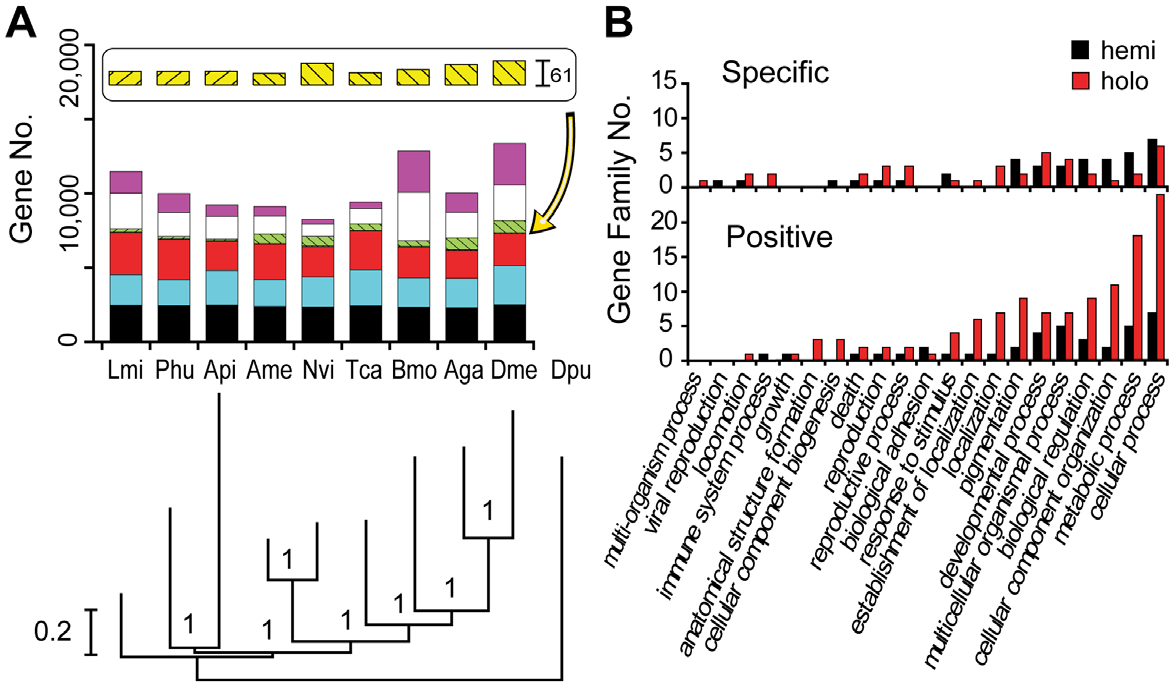
Comparative genomics and evolutionary lineage of insects. A. Insect gene orthology. The gene repertoire in nine insects: locust (LMI), body louse (PHU), and pea aphid (API) (Hemimetabola); fruit fly (DME), mosquito (AGA), silkworm (BMO), red flour beetle (TCA), honeybee (AME), and wasp (NVI) (Holometabola) were compared. Bar color indicates different orthologous relationships. Black: single-copy genes conserved in all insects (allowing one loss) (bottom). Blue: multiple-copy orthologs conserved in all insects (allowing one loss). Red: other orthologs presented in at least one hemimetabola and one holometabola. The striped boxes show hemimetabolous and holometabolous lineage specific orthologs, which were divided into genes conserved in all species in one lineage (lower yellow boxes, enlarged with an arrow) and those conserved in at least two species in the lineage but not all (upper green). White: partial homology but no orthology classified. Purple: species-specific genes. The phylogenetic tree was constructed using water flea (DPU) as outgroup, with numerals indicating estimated posterior probability. B. GO classification of hemimetabolous- (hemi) or holometabolous- (holo) specific gene families and gene families that underwent positive selection exclusively in hemi or holo insects.

To further validate the locust transcriptome and to assess gene orthologous relationships with other insects, we conducted a comparative genomic analysis utilizing 9 insect species: locust, body louse, and pea aphid (which are hemimetabolous insects); and fruit fly, red flour beetle, honeybee, wasp, silkworm, and mosquito (holometabolous insects) (Figure 1A). We identified 27,319 of our locust transcripts as having homology in public databases (Figure S2 and Table S4) and identified 11,490 of these transcripts as locust protein coding genes. Among the protein coding genes identified in the *de novo* locust transcriptome, about 39% were orthologs conserved in all insect species, including 2,464 single-copy orthologs and 2,063 multiple-copy orthologs. We also found 34 orthologs specific to hemimetabolous insects that were present in all three Hemimetabola.

Additionally, 1,787 transcripts similar to transposable elements, and 6 transcripts showing high sequence similarity to non-coding RNAs (Table S5) were identified. We also found 19,468 transcripts (>300 bp) that can neither be annotated by any known proteins nor be predicted by ESTScan [29], and 2,960 of them were also supported by locust ESTs, indicating that the locust transcriptome is a potential resource to explore locust functional non-coding RNAs.

### Genomic divergence between hemimetabolous and holometabolous insects

To determine the effectiveness of transcriptome data for conducting genome-scale phylogenies, we included our locust transcriptome data with genome data from the eight other insects species listed above to construct a phylogenic tree, based on 274 of the most highly conserved 1∶1∶1 orthologs (Figure 1A). Consistent with previous studies [30], [31], all holometabolous orders clustered together, with Hymenoptera as the most basal of Holometabola; two members of the Paraneoptera clade (aphid and body louse) formed a sister group relationship with the Holometabola; and locusts, members of the Polyneoptera clade, were more distantly related to all other insects. Our results provide evidence supporting the assumption that the Hemimetabola is a paraphyletic group and the holometabolous insects originated from hemimetabolous ancestors [31], [32].

To study genomic divergence between hemimetabolous (incomplete metamorphosis) and holometabolous (complete metamorphosis) insects, we identified lineage dependent features of gene sets based on the following two characteristics: presence or absence of genes and gene families, and positive selection. We found that 34 and 21 gene families are Hemimetabola and Holometabola specific, respectively (Table S6); 21 and 54 gene families undergo positive selection exclusively in Hemimetabola and Holometabola, respectively (Table S7). GO classification showed that the divergence of these families encompassed several functional categories, including cellular process, metabolic process, cellular component organization, biological regulation, multicellular organismal process, developmental process, pigmentation, etc (Figure 1B). 18 genes were related to development. The origin of complete metamorphosis brings great changes to insect embryonic development [33], and results in a distinctive pupal stage, making the larva and adult to live in different environment, and thus can avoid potential competition between needs for growth and for reproduction. We found the gene *lack*, a regulator of the dpp signaling pathway in embryonic dorsal-ventral patterning [34], undergoes positive selection exclusively in Holometabola. Among the 18 genes, we also found 4 genes associated with nervous system development [35], 2 genes participating in eye development, 4 genes related to germ cell development and one gene related to the cuticle (Table S8). These findings provided possible candidates for adaptation in the origin of insect metamorphosis.

### Global gene expression

Given the destructive impact of the gregarious phase of the locust, locust phase change has been a primary focus of research on locust biology. We used our *de novo* transcriptome to monitor transcript profiles under the interaction effects of phase and development. Gene expression levels were measured through short reads mapping in RPKM [21] adjusted by a normalization factor [36]. According to *de novo* transcriptome features, the standard uniquely mapped reads approach was adjusted by constructing sequence clusters before reads mapping. Expression measurements were validated by real time PCR experiments (Figure S3).

**Figure 2:**
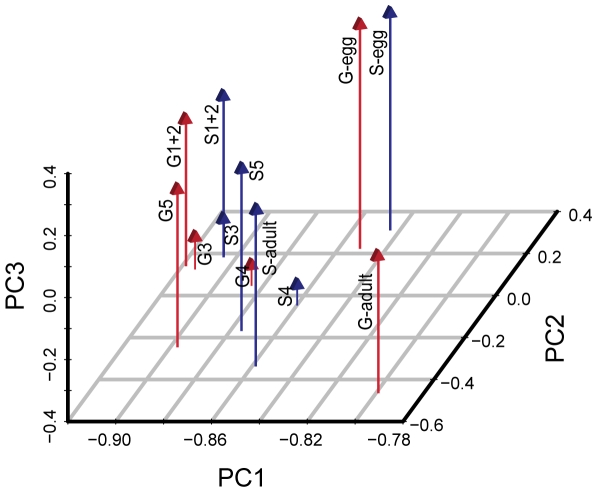
PCA analysis of global gene expression of two locust phases and all developmental stages. PCA: Principal component analysis. G-egg, G1+2, G3, G4, G5, G-adult, S-egg, S1+2, S3, S4, S5, S-adult represent the egg, the 1^st^ and 2^nd^ instar, the 3^rd^ instar, the 4^th^ instar, the 5^th^ instar, and adult of the gregarious locust and the solitary locust, respectively.

Principal component analysis (PCA) revealed that gene expression differences between the developmental stages are much greater than those between the two phases (Figure 2). The first three principal components (PCs) accounted for 88% of the variation observed (Figure S4), with PC1 accounting for the majority (77%). Both phases in each developmental stage consistently clustered together, indicating that gregarious and the solitary locusts have similar developmental processes. The proportion of differentially expressed transcripts between the two phases (FDR<0.01, fold-change>2) increased with the PC1 score (Table S9), indicating that phase differences were mainly reflected in PC1, although PC1 primarily reflected an interaction effect of phase and development. The twelve libraries were arranged on the PC2 axis in developmental order from egg to adult, indicating that PC2 was primarily associated with differences in development. PC3 was not correlated to phase and seemed to reflect some unique features of the 3^rd^ and the 4^th^ instar stage.

### Expression differences between stages

To specifically identify genes that affect the development process, we performed a series of genome-wide expression profiling comparisons to examine gene activity changes between the egg, nymph, and adult stages, without considering phase. By comparing all other post-embryonic stages to the egg, a hierarchical clustering of differentially expressed transcripts (DETs) (FDR<0.01, fold-change>2) generated two main clusters: transcripts up-regulated in the embryonic stage, and transcripts up-regulated in post-embryonic stages (Figure S5), which included 1,604 exclusively embryonic up-regulated transcripts and only 371 exclusively post-embryonic up-regulated transcripts. These results reveal that for the majority of transcripts, the expression differences between egg and all other post-embryonic stages were most homogenous among all post-embryonic stages, and that a greater number of unique genes may be required for embryogenesis than for post-embryonic development. Both the embryonic and post-embryonic clusters were enriched with members of distinct pathways and GO categories (Figure S5).

Pairwise comparisons of each immature stage to the adult stage did not reveal uniformity in expression differences. Hierarchical clustering of DETs showed that the expression difference pattern of 3^rd^ instar vs adult and 4^th^ instar vs adult were similar. The 5^th^ instar and the adult were also highly similar (Figure S6). Enrichment tests revealed that pyruvate metabolism, oxidation reduction, and organelle membrane were enriched in adult development, while starch and sucrose metabolism, pattern binding, carbohydrate binding, and cuticle constituent were enriched in immature development.

### Expression differences between phases

**Figure 3:**
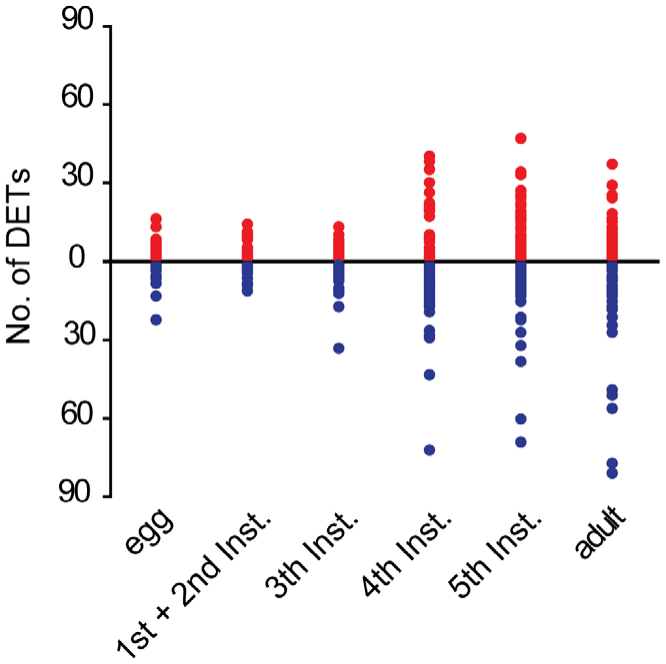
Increase of differentially expressed transcripts between two phases as locusts develop. Up-regulated (red) or down-regulated transcripts (blue) (FDR<0.01, fold-change>2) generated by pairwise comparisons of the gregarious phase vs the solitary phase in egg, 1^st^ and 2^nd^ instar, 3^rd^ instar, 4^th^ instar, 5^th^ instar, and adult were enriched according to their KEGG annotation. Numbers of DETs in all 49 enriched (p<0.01) pathways were plotted. The Y axis is the number of DETs and X axis is six developmental stages.

**Figure 4:**
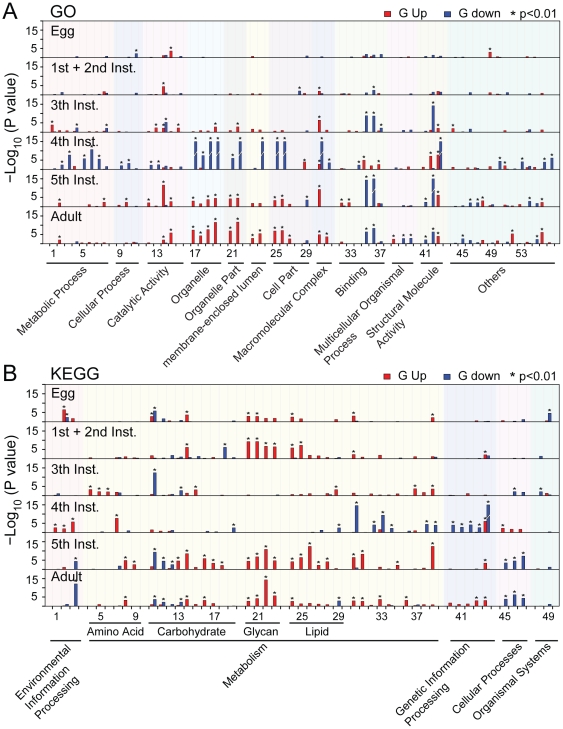
Phase differences in all developmental stages. Enriched (p<0.01) GO categories (A) and KEGG pathways (B) of up-regulated (red) or down-regulated transcripts (blue) (FDR<0.01, fold-change>2) generated by pairwise comparisons of the gregarious phase vs the solitary phase in egg, 1^st^ and 2^nd^ instar, 3^rd^ instar, 4^th^ instar, 5^th^-instar, and adult, and their functional classification. The Y axis is the -log_10_ transformation of the p-value calculated in the enrichment test. The detailed name lists of GO categories (A) and KEGG pathways (B) are given in Table S10 and S11.

Monitoring and comparing transcript profiles between the two phases from various developmental stages showed a sharp rise in phase differences during the 4^th^ instar and then maintained those levels in all following stages (Figure 3 and Figure S7). In the egg stage, the two phases mainly differed in a few specific metabolic pathways (Figure 4). In the gregarious egg, higher activity was seen in galactose, sphingolipid, glutathione, and some glycan metabolism pathways, hydrolase activity, and substrate-specific transporter activity, while cytokinetic process and circadian rhythm was more active in the solitary phase egg. In the 1^st^ and 2^nd^ instar, the phase differences observed in the egg stage remained the same, indicating that the 1^st^ and 2^nd^ instar are still under the influence of parental effects. The 1^st^ and 2^nd^ instar stages also showed the first appearance of differences in oxidoreductase activity, and increases in the glycan and lipid metabolism pathways. Phase differences in the 3^rd^ instar deviated somewhat from those in egg, and 1^st^ and 2^nd^ instars, showing a shift to amino acid metabolism and an increase in various functional categories, indicating a transition in the 3^rd^ instar from the influence of parental effects to the self sense of environmental effects.

The most distinct phase differences in gene expression appeared during the 4^th^ instar stage. Enrichment tests and functional classification showed that the functional categories of metabolic process, intracellular structure, and genetic information processing were more active in the solitary 4^th^ instar, and environmental information processing pathways were more active in the gregarious phase 4^th^ instar, but the functional categories of intracellular structure and some GO terms associated with binding and structural molecule activity were completely reversed in the subsequent 5^th^ instar and adult stages (Figure 4). The 5^th^ instar and adult stages exhibited very similar phase differences: functional categories of intracellular structure, catalytic activity, lipid and glycan metabolism pathways were more active in gregarious locusts while pathways associated with cellular processes were more active in solitary locusts in the two stages, but phase differences associated with multicellular organismal process were unique in adults.

We suppose that the common phase differences exhibited by 5^th^ instar and adult stages represent stable physiology, endocrinology and metabolism differences between the two extreme phases, and the 4^th^ instar stage seems to be a turning point in the process of forming these phase differences. The marked differences seen in the 4^th^ instar stage indicated that detailed analysis of deep sequencing data of the 4^th^ instar would likely provide the most informative data for identifying molecular changes related to phase change.

### Phase marker genes

Of the annotated transcripts, we found 194 transcripts specifically expressed in the solitary or the gregarious locusts (expressing in at least 5 developmental stages in one phase but having no expression in at least 4 stages in the other phase) and 48 transcripts whose expression levels were not affected by development (not considering eggs), but exhibited stable difference between two phases (coefficient of variability <0.3 within gregarious phase group or solitary phase group; mean ratio of the two groups >1.5; p-value for t test of the two groups <0.05). We considered these 242 transcripts (Table S12) as candidates for phase marker genes. Most phase marker gene candidates were associated with metabolism and biosynthesis (Table 2), since the metabolic change is a reflection of upstream events. Choline/ethanolamine kinase and insulin receptor tyrosine kinase were found to be possible solitary markers.

**Table 2:** Functional classification of phase marker gene candidates according to KEGG annotation

KEGG Classification	Specific			Stable Expression Difference Between Phases		
	S	G	Total	S	G	Total
Metabolism	14	27	41	5	9	14
Genetic Information Processing	7	10	17	2	9	11
Environmental Information Processing	2	8	10	0	7	7
Cellular Processes	8	12	20	0	3	3
Unclassified	1	0	1	1	1	2
Total	32	57	89	8	29	37

Interestingly, we found several genes that can be related to neural functions and behavioral plasticity: (1) *cask* is involved in regulation of neurotransmitter release [37] and was a gregarious marker gene candidate; (2) *Fmr1*, whose deficiency sometimes is accompanied by autism-like behavior [38], can function in adult neurogenesis [39] and showed constitutively higher expression in gregarious locusts; (3) *Malvolio*, which is required for fly taste behavior [40], constitutively higher expressed in gregarious locusts and may be related to differences in feeding behavior of two phases. In the gregarious marker gene candidates, we also found the nuclear receptor, its interacting protein mediator complex, guanylate cyclase and some other genes that function in signal transduction, indicating a special need for signal transduction of gregarious locusts.

### Divergence of the fourth instar nymphs between phases

Deep-sequencing the two different phases of the 4^th^ instar locust generated 158 million reads for the solitary 4^th^ instar (S4) and 218 million reads for the gregarious 4^th^ instar (G4). KEGG and GO analysis showed that 8,520 G4 up-regulated transcripts (FDR<10^−5^, fold-change>2) were enriched in several functional categories: environmental information processing pathways, transcription factor activity, multi-organism process, etc. In contrast, the 5,772 S4 up-regulated transcripts (FDR<10^−5^, fold-change>2) were enriched primarily in the functional categories of metabolic process and intracellular structure (Figure S8 and S9).

**Figure 5:**
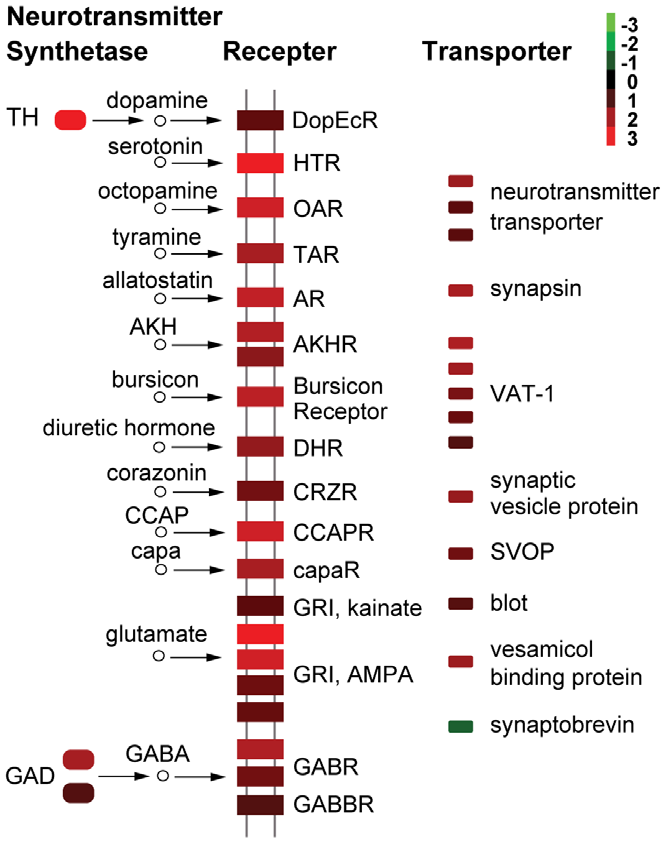
Differential expression of neurotransmitter receptors, synthetases, and transporters between two phases of 4^th^ instar nymphs. The log_2_-transformed ratio of (RPKM in G4)/(RPKM in S4) for each transcript was used. Red and green colors indicate up- and down-regulated transcripts, respectively. Abbreviations: DopEcR, HTR, OAR, TAR, AR, AKHR, DHR, CRZR, CCAPR, capaR and GRI denote the receptor of dopamine, serotonin, octopamine, tyramine, allatostatin, adipokinetic hormone (AKH), diuretic hormone, corazonin, cardioacceleratory peptide (CCAP), capa peptide and glutamate, respectively; GABR and GABBR denote the type A and type B receptor of gamma-Aminobutyric acid (GABA), respectively; TH: tyrosine hydroxylase; GAD: glutamic acid decarboxylase; VAT-1: vesicle amine transporter 1; SVOP: synaptic vesicle 2-related protein.

The most enriched pathway of the G4 up-regulated transcripts was the neural pathway involving neuroactive ligand-receptor interactions (Figure 5 and Figure S8). We found high expression in G4 of the receptors for dopamine, serotonin, octopamine, tyramine (monoamines); allatostatin, adipokinetic hormone (AKH), bursicon, diuretic hormone, corazonin, cardioacceleratory peptide (CCAP), and capa peptide (neurohormones and neuropeptides); and glutamate and GABA (amino acids) (Figure 5). We also found differential expression in neurotransmitter synthetase and synaptic transport components (Figure 5) that are important for neural pathway function. Rate-limiting enzymes in the synthesis of dopamine and GABA were significantly up-regulated in G4. The prevalence of the significantly higher expression of neurotransmitter receptors, synthetases, and synaptic transport components in G4 indicated that neurotransmitters and neuromodulators are likely to play important roles in locust phase change.

**Figure 6:**
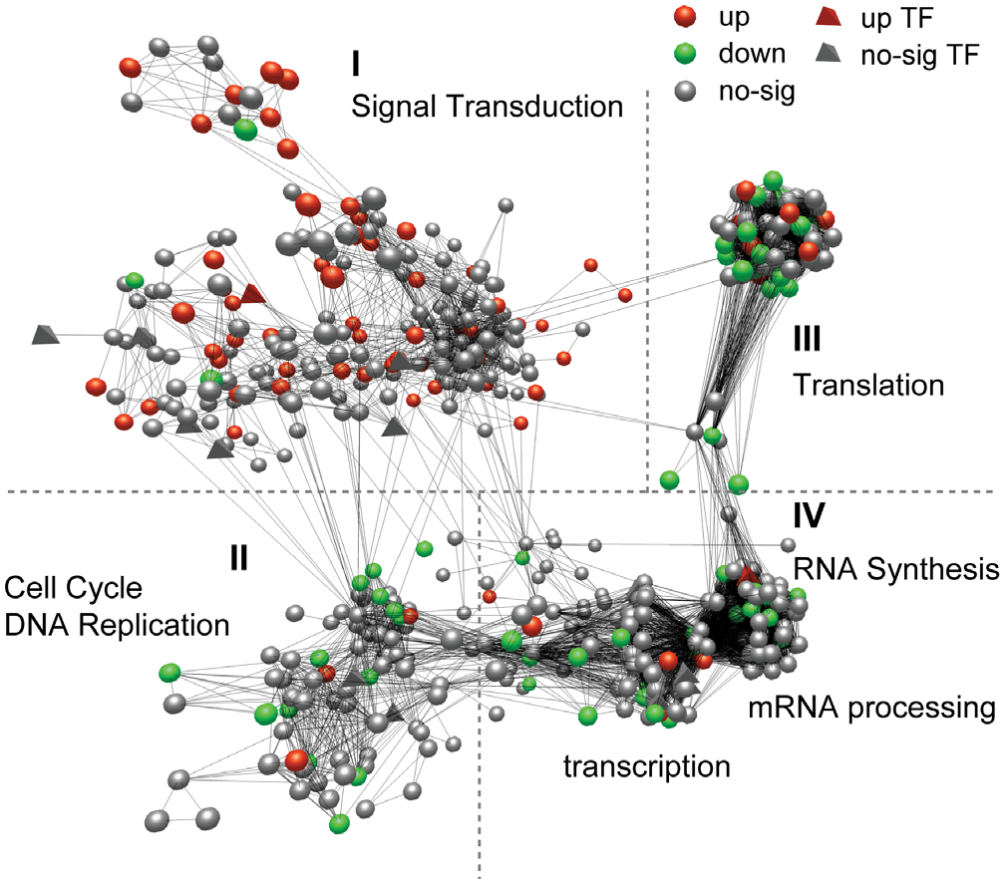
A functional network of differentially expressed locust orthologs between phases in the 4th instar stage. This network was inferred from an existing set of *Drosophila* interaction networks in Reactome based on locust orthologs to *Drosophila* genes. A node (triangle for a transcription factor (TF) and sphere for a protein) indicates a locust orthology to a *Drosophila* gene. An edge represents a protein-protein interaction inferred from the existing *Drosophila* interaction networks. Red, green and gray colors denote being up- or down-regulated or showing no significance in G4 vs S4, respectively. BioLayout was used to produce network graphs [74].

To better understand how neurotransmitter activity is regulated in locust phase polyphenism, we constructed locust functional networks inferred from an existing set of *Drosophila* interaction networks in Reactome, a knowledgebase of biological pathways and processes [41], and mapped our differentially-expressed locust orthologs into these networks. We identified one major network and several small networks spatially separated. The major network contained 564 nodes (genes) and 6,593 edges (interactions), accounting for a majority (143, 62%) of the mappable, differentially-expressed orthologs (Figure 6) with related functional relevancy. There were four functional groups within this network.

Almost all of the G4 up-regulated orthologs were present in the signal transduction group (Group I) in the major network, and were primarily associated with signaling by G protein-coupled receptors (GPCR), Rho GTPases, MAPK, Notch, and neurotrophins; and in axon guidance (Table S13). Many of these signal pathways are involved in the regulation of neurotransmitter activity [42], [43]. GPCR signaling is central to this signal transduction cascade. Most of the neurotransmitter and neuromodulator receptors are GPCRs, and many downstream effectors of GPCRs are also up-regulated in G4 (Table S14).

In addition, we identified a small network of G4 up-regulated nuclear receptors, an important class of transcription factors (TFs), and their interacting proteins (Figure S10). The GO term of transcription factor activity was also enriched in G4 up-regulated transcripts. The locust transcriptome contained orthologs to 255 *Drosophila* site-specific TFs (FlyTF) [44], 58 of which were G4 up-regulated and 18 down-regulated.

Most G4 down-regulated orthologs were present in the three other functional groups in the major network: group II (translation), made up of ribosomal proteins and translation initiation factors; group III, including proteins functioning in cell cycle and DNA replication; and group IV (RNA synthesis), consisting of two sub-groups, IV-1 (transcription) with RNA polymerase II and general transcription factors, and IV-2 (mRNA processing) with splicing related proteins. These data were consistent with the GO and KEGG enrichment results, indicating that solitary 4^th^ instar locusts are more active in genetic information processing and biosynthesis than the gregarious ones.

## Discussion

### Sequencing and assembly strategy

The design of *de novo* transcriptome works, such as library construction, the amount of sequencing and assembly strategy, has great influences on the assembly quality [45], [46]. For *de novo* transcriptomes, to discover more genes and sometimes also to investigate gene expressions, multiple libraries from different samples would be constructed. How to arrange the amount of sequencing for each sample is important when the total amount is limited. We found that pooling all reads from various samples has a negative effect on sequence elongation. This may be caused by the introduction of new alternative splicing, repeats and other uncertain factors when pooling reads from different samples. So we recommend assembling samples separately and only focusing on a few of samples, since the quality of each assembly is the most important and should be guaranteed by sufficient amount of sequence data. We demonstrated that this strategy substantially improved the assembly results. To apply *de novo* transcriptome to genome-wide gene discovery, one of the impediments is the uncertainty of experiment design. Our optimization of experiment designs as well as the success of this locust transcriptome will facilitate further application of *de novo* transcriptomes.

Investigating gene expressions in other samples can be carried out by sequencing each in one lane. Comparing transcript reads counts between deeply sequenced G4 and S4 only found a few of transcripts unique to one sample (Figure S11). About half of total transcripts can be detected in any one of the lanes sequenced for G4 or S4 (Figure S11), and increasing the number of lanes counted leads to slow increase of transcripts detected (Figure S12), indicating that sequencing in one lane is efficient to do expression profiling and deep sequencing data are the most informative.

### A locust core gene set

This study represents the largest *de novo* transcriptome to date. Our aim is to develop a comprehensive and representative core gene set of the migratory locust. We considered the locust protein coding genes we generated as a locust core gene set for the following reasons. We have performed 21 Gb of Illumina sequencing. The large amount of data provided the first guarantee for reaching a high coverage of locust genes. The 11,490 locust protein coding genes were identified after removing redundancy by self-to-self similarity search, of which 5,478 have a mutual-best-hit in the fruit fly (>40% of fruit fly genes in flybase release 5.8). Considering the similarities shared by insects in gene content, for example the pea aphid which shares 30–55% of its genes with other sequenced insects [47], a similar extent has been achieved in our locust transcriptome study, indicating that the locust transcriptome has a high coverage of locust gene content. In addition, in the analysis of genes conserved in 9 sequenced insect species, inclusive of locust, the number of the widespread conserved insect genes is in a range of 4,000 to 5,500 across all species, indicating that conserved insect genes are highly represented in our locust transcriptome.

The high coverage of locust gene content enriches the genetic resources for hemimetabolous insects. Subsequent genome-scale phylogenies and evolutionary analysis on genomic divergence between hemimetabolous and holometabolous insects add to our understanding of the origin of insect metamorphosis.

### Compare to previous locust ESTs

Due to the high throughput of RNA-seq and the large amount of sequencing as discussed above, this locust transcriptome has a much higher coverage of locust gene content than previous ESTs does. Compared to the ESTs, nearly half (1,994, 44%) of the widespread conserved insect genes were first identified in this locust transcriptome, and a half (1,834, 59%) of other types of conserved insect genes were also first identified. The locust transcriptome also newly covered 4,473 more fly genes, placing locust researches onto a much more wide knowledge scope.

More than half of transcriptome DETs were not detected by previous EST sequencing. Of those detected, 287 were also included in the EST DETs [14]. GO classifications of the overlapping DETs and DETs specific to transcriptome or ESTs are similar (Figure S13). The GO term of synapse and synapse part are unique in the transcriptome DETs, with most members first detected by transcriptome sequencing, providing good opportunities to study brain function in the locust phase change.

Despite the advance in gene content coverage, the ability in gene expression profiling is also much advanced by RNA-seq. Different from EST sequencing which is a cloning-based amplification approach, RNA-seq is PCR-based amplification, eliminating the cloning step and possible host-related biases introduced by cloning [16]. Besides the high throughput of RNA-seq overcomes the shortcoming of low redundancy of sequencing reads in EST data, which makes EST data not suitable for quantitative measuring transcript abundance [16], [18]. Additionally, compared to microarray method, RNA-seq can overcome the limitation of hybridization background and different hybridization properties of different probes [48].

Additionally, the previous EST analysis focused on the 5^th^ instar locusts and studied gene differential expression between phases of different organs, while this study focused on the relationship between phase and development.

### Effects of development on phase differences

We first discovered how divergence between the two phases in gene expression generally progresses as locusts develop. Firstly, the number of DETs between phases increases as locusts develop, reflecting a cumulative effect on phase traits. However, it is not a gradual increase. A sharp rise appears in the 4^th^ instar stage (Figure 3 and Figure S7), indicating the complexity of the cumulative effect. Secondly, pathways and GO categories associated with DETs in each developmental stage do not remain the same (Figure 4). Resemblance of phase differences is observed between egg and the 1^st^ and 2^nd^ instars, indicating a parental effect. Phase differences in the 5^th^ instar and adult stage are highly similar, but the 3^rd^ instar and 4^th^ instar are unique.

Lastly, the phase differences in the 4^th^ instar stage is very different from the 5^th^ instar and adult stages, especially the functional categories of intracellular structure and some GO terms associated with binding and structural molecule activity which are completely reversed (Figure 4). This unique pattern in the 4^th^ instar stage may indicate that switches in phase-related events occur at the physiological or molecular level during that period. Some studies on desert locusts also implied there might be a switch in phase-related events during the intervening time period between the 3^rd^ and the 5^th^ instar stage. Projection interneurones (PNs) from the gregarious desert locusts were found to be more sensitive to odour stimuli than the solitary ones in the 3^rd^ instar [49], whereas the opposite was true in the 5^th^ instar [50]. This reversal pattern was also found in the number of antennal sensilla [51].

### Divergence in biological investment of two phases

Our work first discovers the phase-dependent divergence of biological investment in the molecular level and highlights related genes and pathways. Both the phase marker gene and the detailed analysis of deep sequencing data of 4^th^ instar locusts showed that gregarious locusts are more active in pathways involved in detecting and processing of environmental information, while solitary locusts are more active in pathways associated with metabolism and biosynthesis. This difference indicates a divergence in biological investment that gregarious locusts invest more on interaction with the environment but solitary locusts appear to have a molecular focus on the maintenance of their existence.

A recent morphological research on brains of the desert locust found that gregarious locusts had larger brains along with higher proportion of midbrain, but solitary locusts had smaller brains with much larger primary sensory neuropiles [52], providing anatomical evidence to the divergence of biological investment of two phases. The anatomical differences are results of long-term phase-dependent gene differential expression but in turn its structural basis. For locusts living in long-term crowded populations, post-embryonic neurogenesis may occur, and may be a reason for the observed brain size differences between phases [52]. Some nuclear receptors are shown to play roles in adult neurogenesis [53], [54]. The highly expression of genes involved in adult neurogenesis may contribute to the process, such as nuclear receptors, their interacting proteins and the gregarious phase marker gene candidate *Fmr1*.

### Framework of locust molecular networks

Complex biological processes, such as the locust phase transition which involves changes in behavior, body color, physiology and even reproductivity, are not likely to be controlled by a single gene but multiple genes that interact in molecular networks. Integration of knowledge from fruit fly provided a framework of locust molecular networks that allowed us to depict differential regulation of gene expression between two phases. Within the framework, based on the higher activity of genes and pathways associated with environmental information processing in gregarious locusts, we propose that locusts respond to crowded population density signals by regulating their neurotransmitter activity, and GPCR signaling pathways participate in this regulation.

The pathway of neuroactive ligand-receptor interactions and the GO term of neurotransmitter binding are one of the most enriched pathways and GO terms of the gregarious 4^th^ instar (G4) up-regulated transcripts, including most neurotransmitter and neuromodulator receptors detected in the locust transcriptome (Figure 5). Many of the neurotransmitters are known to mediate phase related behavioral and physiological traits: serotonin, a neurotransmitter, can induce gregarious behavior in the desert locust [11]; dopamine was demonstrated in a recent work to be strongly linked with gregarious behavior and body color of the migratory locust (Kang L, et al. unpublished) and interestingly the dopamine receptor indentified here is *DopEcR*, which can also be activated by insect ecdysteroids [55]; octopamine promotes aggressive behavior of fruit fly [56]; AKH induces the release of lipid from fat body to fuel flight during locust migration [57]; and corazonin induces dark color in locust [58]. Most of the neurotransmitter receptors are G protein-coupled receptors (GPCR) and many downstream effectors of GPCRs are up-regulated in G4 (Table S14). In the functional network of G4 DETs, GPCR signaling is central to the signal transduction sub-group (containing most G4 up-regulated transcripts) (Figure 6 and Table S13), which includes pathways involved in regulating neurotransmitter activity. For example, Gα12 signaling and Rho GTPases can regulate neurotransmitter release at synapses [42], and neurotrophin signal pathway can modulate synaptic plasticity including neurotransmitter receptor activities [59].

Increasing of population density elicits striking changes in locust behavior and body color. Receiving and processing external stimuli in the population are crucial in this process. Neurotransmitters may play a role as the endogenous effectors that intervene between the external stimuli and gene expressions. Above signal transduction networks may be responsible for coordinating downstream gene expressions that mediates the forming or transition of phase traits.

Taken together, our locust transcriptome provides data to further our understanding of locust biology, especially with regard to phase transition mechanisms and pest management. The pathways and genes identified here that are potentially important for locust development and subsistence may provide useful pest management targets. Those associated with phase transition, especially GPCRs, which are one of the most popular classes of drug targets in pharmaceutical industry, will aid in developing novel strategies to achieve successful control of locust outbreaks in the future.

## Materials and Methods

### Insects

Gregarious locusts and solitary locusts were reared as previously described [14]. Locusts were collected in whole bodies (remains in the alimentary canal were removed), and 15 to 20 individuals were collected for each sample.

### RNA-seq library preparation and Illumina sequencing

Total RNA was extracted using TRIzol reagent (Invitrogen) and treated with RNase-free DNase I. Poly(A) mRNA was isolated using oligo dT beads. First-strand cDNA was generated using random hexamer-primed reverse transcription, followed by synthesis of the second-strand cDNA using RNaseH and DNA polymerase I. Then, single-end and paired-end RNA-seq libraries were prepared following Illumina's protocols and sequenced on the Illumina GA II platform.

### Assembly

*De novo* assembly of the short reads was performed using SOAPdenovo [25] as follows. K-mers 19, 21 and 23 were tested and k-mer 23 was used at last because of its better performance.

We filtered low quality short reads and corrected the reads according to the 23 mer frequency, since the de Bruijn graph algorithm is sensitive to the sequencing error. Then we used the overlap information in the short reads to construct contigs with high coverage. In this step, we first broke down all reads into 23 mers which were used as nodes to construct the de Bruijn graph and two reads, whose overlap were 22 bp, were connected. To reduce the graph complexity, we filtered the “tips” which were shorter than 50 bp, removed low coverage dead ends in the graph, and then merged the bubbles which represent parallels. Following these steps, we assembled the short reads into contigs. We then realigned the short reads onto the contigs and estimated the distance and relation of the two contigs using the pair-end linkage and insert size information. Unreliable linkages between two contigs were filtered and the remaining contigs with compatible connections to each other, and having at least three read-pairs, were constructed into scaffolds. The last step is to close gaps in the scaffolds. We gathered the paired-end reads with one end mapped to the contigs and another end located in the gaps and performed local assembly with the unmapped end to extend the contig sequence in the small gaps in the scaffolds. CAP3 [27] was used (with default parameters) to reduce redundancy and also to combine scaffolds and single-end contigs in the separate assembly.

### Annotation

To annotate the locust transcriptome, we performed a BLAST search against the non-redundant (NR) database in NCBI, SWISS-PROT, KEGG and COG with an e-value cut-off of 1e-5. We annotated the motifs and domains using InterPro [60]. Gene Ontology terms were assigned by Blast2GO [61] through a search of the NR database.

### Comparative Genomic Analysis

#### Locust protein coding genes

Protein coding gene set was identified by analyzing Blastx results against NR database in NCBI with e-value <1e-5 and only those sequences whose best hit belonged to the arthropod species were extracted. Low quality sequences, transposable element and putative contamination sequences were filtered (see Methods S1). Putative alternative splicings and redundancy were also filtered as follows.

Remaining sequences were clustered based on sequence similarity with a criteria of overlap ratio >60% to any sequences and identity >0.96. The longest sequence in a cluster was selected to represent the gene where they transcribe. These criteria were set up according to the overlap ratio between alternative splicing pairs calculated using *Drosophila*'s gene annotation information. We found that the quantile of overlap ratio is 0.0092, 0.56, 0.81 and 0.92, respectively. To filter the influence of alternative splicing on subsequent orthology analysis more tightly, we set up the overlap ratio as >60%. Self-to-self similarity search results of different overlap ratios were listed in Table S15. Identity >0.96 were used to discriminate paralogy from the alternative splicing, because the overlapping regions of the former do not have such high identity due to mutation.

#### Orthology

After sequence preparation, gene families were constructed and orthologous relationships were identified by TreeFam [62] method (see Methods S1).

#### Phylogeny

The phylogeny tree was reconstructed from the 274 most highly conserved single-copy gene families (present in all species, identity >70%, gap <0.3 in alignment). A Bayesian Markov chain Monte Carlo method [63] was implemented using a CAT-BP model to construct the phylogenic tree (see Methods S1).

#### Positive selection

An improved branch-site model A [64], [65] was used to extrapolate the sites under positive selection (dN/dS>1 in one lineage and dN/dS < = 1 in other lineages). The outgroup was removed prior to analysis. Positive selection criteria: p-value <0.05; and has at least one positive selected site with Bayesian empirical Bayes posterior probability >0.95.

### Expression profiling pipeline

This pipeline includes following three parts and is freely available at http://www.ipm.ioz.ac.cn/kang/webpages/locusttranscriptome.html.

#### Gene expression value measurement

Gene expression profiling was measured by mapping reads to assembled sequences using SOAP [66]. The most widely used approach is to count uniquely mapped reads. For transcriptomes having reference genome, several methods have been provided to deal with isoforms [67], [68]. But in the case of *de novo* transcriptome lacking reference genome and isoform information, counting uniquely mapped reads are affected by alternative splicing and redundancy. Therefore, we utilized sequence similarity information among transcripts to construct sequence clusters by self-to-self similarity search. The criteria are overlap ratio >80% to any sequences and identity >0.96. Here, the overlap ratio is set up according to the median of *Drosophila*'s gene annotation information mentioned above. To avoid including real multiple mapped reads that map to different genes, a more stringent criterion of >80% overlap was selected to restrict the number of transcripts included in a cluster and thus restrict reads involved by this adjustment (Table S15). After sequence cluster constructed, for single sequences with no cluster, only uniquely mapped reads were counted; for sequences within a cluster, the mapped reads hit on multiple sequences that all belonged to the same cluster were also counted.

Then, the RPKM value for each transcript was measured in reads per kilobase of transcript sequence per million mapped reads [21]. To exclude the bias caused by different RNA output between samples, we adopted a TMM (trimmed mean of M values) method to calculate a normalization factor introduced by Robinson [36], using calcNormFactors function in edgeR package [69]. S-egg was selected as the reference sample. The effective library size was calculated by multiplying the square root of the estimated normalization factor with the original library size. The effective size was used to adjust RPKM values in following analysis.

In order to avoid the influence of sequencing depth and to keep single-end libraries and deeply sequenced paired-end libraries consistent, when analysis involved both kind of libraries, we use one lane and one end that were randomly selected to represent the paired-end library and treat the data as single-end 35 bp reads, as for reads in other single-end libraries.

#### Differentially expressed genes

We modified Audic's [70] method to analyze differential expression. Audic's method was used originally for pairwise comparisons of cDNA libraries. The formula to calculate the significant p-value is defined as follows.
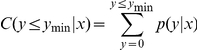


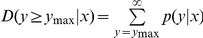
In which,
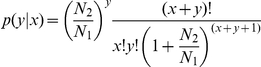



Where, N1, N2 indicate the sampling size of two libraries, and x, y indicate the number of times that a transcript is sampled in the libraries. When we apply this method to the RNA-seq data, the total mapped read counts of two libraries can be used to represent the sampling size (N1, N2) and the mapped read counts of one transcript in the two libraries can be used to represent the number of times that a transcript is sampled (x, y). However, for the reason that in RNA-seq experiments the expected read counts of a given transcript is proportional to its abundance multiplied by its length [21], [71], [72], x, y represented by the mapped read counts of a transcript can not reflect the real expression level of transcripts. Therefore, we defined x and y here as mapped reads per kilobase of a transcript to avoid bias of sequence length. In addition, as stated in Audic's paper, more reliable inferences will be made on transcripts corresponding to larger absolute frequencies detected and the absolute frequencies is proportional to the sampling size N in a given expression level [70]. Thus, in a serial of pairwise comparisons, in a given fold change of expression levels, two transcripts in library pairs with higher sampling sizes will have greater statistical weights for detecting differential expression, compared to those in library pairs with lower sampling sizes. Besides, in RNA-seq data the expected mapped reads of a transcript in a library is proportional to the sampling size of the library. Therefore, when results of a serial of pairwise comparisons are to be compared, we normalize N1, N2, x and y to the smallest library to avoid the influence of different sampling size among different library pairs.

The FDR cutoffs to select differentially expressed transcripts were set as: FDR<0.01 for a serial of pairwise comparisons using libraries sequenced in one lane; and FDR<10^−5^ for analyzing deeply sequenced libraries G4 and S4 alone. A more stringent FDR cutoff was set for deeply sequenced libraries, for the above reason that higher sampling sizes will have greater statistical weights for detecting differential expression.

#### Enrichment analysis

Enrichment analysis for the supplied gene list was carried out based on the algorithm presented by GOstat [73], with the whole locust transcriptome set as the background. The p-value was approximated by the chi-square test. Fisher's exact test was used when any expected value of count was below 5, which will make the chi-square test inaccurate.

### Principal component analysis

Prior to PCA analysis, expression levels in RPKM were normalized with zero mean and unit variance and transcripts expressed in fewer than six libraries were filtered. PCs, PC variances and PC scores (gene loadings) were calculated using eigen function in R.

### Real time PCR

SYBR Green real time PCR was performed to validate RNA-seq data. Whole bodies of insects were used and 5 individuals were collected for each sample. The standard curve method was used, with 18S as endogenous control. There were three biological and technical replicates respectively. The data were analyzed by SPSS. Transcripts for validation were selected randomly and primers were listed in Table S16.

## Supporting Information

Methods S1 **Supporting methods.**
(DOC)

Figure S1 **Evaluation of assembled sequences.** A. Assembled transcripts were compared with the evaluating EST set (11498 ESTs, with >90% coverage by at least 2X Illumina reads). B. Assembled transcripts were compared with full length cDNAs available in GenBank, and ORFs confirmed experimentally in our laboratory. C. An example of full length cDNA covered by assembled transcripts and ESTs. D. Five transcripts were validated by full length cDNA clones using RACE PCR. Y axis in the left (green bars) is the length of validated transcripts and y axis in the right (red points) is the ratio of validated regions.
(DOC)

Figure S2 **Similarity search results against non-redundant (NR) database in NCBI.** A. E-value distribution. B. Organism distribution.
(DOC)

Figure S3 **Real time PCR validation.** * represents gene differential expression between two samples with significance. Real time PCR data were analyzed by independent two sample t test (P <0.05 for significance) and were represented by means ± SD. RNA-seq data of G4 and S4 were measured in RPKM. Transcripts for validation were selected randomly. Their annotations were listed according to the order in the figure as follows: VAT-1; Rho GTPase; Dorsal switch protein 1; ecdysone 20 hydroxylase; lipophorin receptor; Basement membrane-specific heparan sulfate proteoglycan core protein; TPR repeat-containing protein; chitinase; similar to CG32104; Mucin-5AC; taxilin alpha; conserved hypothetical protein.
(DOC)

Figure S4 **Cumulative variance of PCs in principal component analysis of the 12 libraries.**
(DOC)

Figure S5 **Embryonic and post-embryonic development of the locust.** Left is the result of hierarchical clustering of differentially expressed transcripts (FDR<0.01, fold-change>2) generated by the pairwise comparison of all post-embryonic stages to the egg. It generated two main patterns. Red stands for up-regulating in post-embryonic stages (stages except the egg) and green stands for up-regulating in embryonic stage (the egg). Right is the KEGG enrichment of these two patterns. The Y axis is –log_10_ transformation of the p value calculated in enrichment test. Hierarchical clustering was performed using Gene Cluster 3.0.
(DOC)

Figure S6 **Adult and immature development of the locust.** Left is the result of hierarchical clustering of differentially expressed transcripts (FDR<0.01, fold-change>2) generated by the pairwise comparison of all immature stages to the adult. It generated four main patterns. Red stands for up-regulating in immature stages (stages except the adult) and green stands for up-regulating in the mature stage (the adult). Right is the KEGG enrichment of these four patterns. The Y axis is –log_10_ transformation of the p value calculated in enrichment test. Hierarchical clustering was performed using Gene Cluster 3.0.
(DOC)

Figure S7 **Number of differentially expressed transcripts (FDR<0.01, fold-change>2) in all development stages.**
(DOC)

Figure S8 **KEGG enrichment for gregarious up-regulated (red) or down regulated transcripts (blue) (FDR<10^−5^, fold-change>2) generated by pairwise comparison of the two deeply sequenced libraries (G4 and S4).** The Y axis is –log_10_ transformation of the p value calculated in enrichment test.
(DOC)

Figure S9 **GO enrichment for gregarious up-regulated (red) or down regulated transcripts (blue) (FDR<10^−5^, fold-change>2) generated by pairwise comparison of the two deeply sequenced libraries (G4 and S4).** The Y axis is –log_10_ transformation of the p value calculated in enrichment test.
(DOC)

Figure S10 **A small functional network in locust transcriptome consisting of nuclear receptors and their interacting proteins.** Red, green and gray colors denote being up- or down-regulated or showing no significance in G4 vs S4, respectively. Triangles represent transcription factors and spheres represent other proteins.
(DOC)

Figure S11 **Number of transcripts detected in different number of lanes in the deep sequencing data and length distribution of transcripts that can only be detected in lanes from G4 or S4.** Sample G4 was sequenced in 16 lanes and S4 in 9 lanes. Reads of each lane from G4 and S4 that can be mapped to transcripts (that means this transcript can be detected in that lane) were recorded. Number of transcripts that can be detected using any N lanes from G4 or S4 was plotted. Group 1: N = 1, that means can be detected using any one lane in G4 or S4, i.e. expressing in all the lanes in G4 or S4; Group 2: N = 2∼8 for G4, N = 2∼4 for S4; Group 3: N = 9∼16 for G4, N = 5∼9 for S4. Red: can be detected only in lanes from G4; blue: can be detected only in lanes from S4; gray with strips: can be detected in lanes both from G4 and S4. On the top is the length distribution of the transcripts unique to one sample.
(DOC)

Figure S12 **Length distribution of transcripts that express in different number of lanes.** Sample G4 was sequenced in 16 lanes and S4 in 9 lanes. Reads of each lane from G4 and S4 that can be mapped to transcripts (that means this transcript expresses in that lane) were recorded. Every histogram represents the length distribution of transcripts that only express in N (the number in yellow strips) lanes in G4 (A) and S4 (B). For example, “1” in (A) represents transcripts that only express in one of the 16 lanes from G4, i.e. can only be detected by using all lanes; “16” in (A) represents transcripts that express in all the 16 lanes from G4, i.e. can be detected by using any one lane. X axis is the number of transcripts and Y axis is the length of transcripts (bp).
(DOC)

Figure S13 **GO classification of the overlapping DETs and DETs specific to transcriptome or ESTs.** WEGO was used to produce graphs. A. The second GO level. B. The third GO level of Cellular Component.
(DOC)

Table S1 **Experimental design and sequence reads generated.**
(DOC)

Table S2 **Assembly statistics of contigs generated by assembly of the pool of all reads (PAAR) and assemblies of reads from G4 and S4 separately.**
(DOC)

Table S3 **Assembly statistics of scaffolds generated by assembly of the pool of all reads (PAAR) and assemblies of reads from G4 and S4 separately.**
(DOC)

Table S4 **Transcriptome annotation by sequence similarity (e-value<1e-5).**
Sequences with length greater than 300 bp were subjected to annotation. InterPro was searched by InterProScan. GO was searched by Blast2GO.
(DOC)

Table S5 **Non-coding RNAs identified by BLASTN in locust transcriptome.**
The following non-coding databases were searched by BLASTN: RNAdb, NONCODE, Rfam, ncRNAdb and fRNAdb. Insect rRNAs were also searched.
(DOC)

Table S6 **Hemimetabolous and holometabolous insect specific gene families.**
LMI: *Locusta migratoria*; PHU: *Pedicularis humanus*; API: *Acyrthosiphon pisum*; NVI: *Nasonia vitripennis*; AME: *Apis mellifera*; TCA: *Tribolium castaneum*; BMO: *Bombyx mori*; DME: *Drosophila melanogaster*; AGA: *Anopheles gambiae*.
(DOC)

Table S7 **Gene families underwent positive selection exclusively in hemimetabolous insects or in holometabolous insects.**
LMI: *Locusta migratoria*; PHU: *Pedicularis humanus*; API: *Acyrthosiphon pisum*; NVI: *Nasonia vitripennis*; AME: *Apis mellifera*; TCA: *Tribolium castaneum*; BMO: *Bombyx mori*; DME: *Drosophila melanogaster*; AGA: *Anopheles gambiae*.
(DOC)

Table S8 **Gene families under adaptation pressures which affect development process.**
(DOC)

Table S9 **Distribution of differentially expressed transcripts (DETs) in PC1.**
(DOC)

Table S10 **The index number in** **Figure 4A** **and the enriched (p<0.01) GO categories of gregarious up-regulated or down regulated transcripts (FDR<0.01, fold-change>2) generated by pairwise comparison of the two phases in egg, 1^st^ and 2^nd^ instar, the 3^rd^ instar, 4^th^ instar, 5^th^ instar, and adult.**
(DOC)

Table S11 **The index number in** **Figure 4B** **and the enriched (p<0.01) KEGG pathways of gregarious up-regulated or down regulated transcripts (FDR<0.01, fold-change>2) generated by pairwise comparison of the two phases in egg, 1^st^ and 2^nd^ instar, the 3^rd^ instar, 4^th^ instar, 5^th^ instar, and adult.**
(DOC)

Table S12 **Candidates for phase marker genes.**
Candidates with clear annotations were listed.
(DOC)

Table S13 **Main signal pathways and events involved in the Group I of the major functional network.**
(DOC)

Table S14 **Differentially expressed genes between the two phases of 4^th^ instar locusts in downstream events of GPCR signaling.**
Up and down means DETs (differentially expressed transcripts) up-regulated or down-regulated in the gregarious 4^th^ instar locusts, respectively.
(DOC)

Table S15 **Statistics of self-to-self similarity search results in different overlap ratios (identity >0.96).**
(DOC)

Table S16 **Primers used in the real time PCR experiment.**
(DOC)
