# Supplementary material for: De Novo Analysis of Transcriptome Dynamics in the Migratory Locust during the Development of Phase Traits
Source: PLoS One. 2010 Dec 30;5(12):e15633. doi: 10.1371/journal.pone.0015633 (PMC3012706; doi:10.1371/journal.pone.0015633)
Supplement: Methods S1 — Supporting methods. (DOC) [file pone.0015633.s001.doc]

# Methods S1

Used data sets

The protein sequences in fasta format and mRNA sequences in GenBank format files of *Acyrthosiphon pisum,* *Nasonia vitripennis*, *Apis mellifera*, *Tribolium castaneum,* and *Anopheles gambiae* are downloaded from NCBI ([www.ncbi.nlm.nih.gov/](http://www.ncbi.nlm.nih.gov/)). The CDS sequences of these organisms are extract from the mRNA GenBank format files. Protein and CDS sequences of *Bombyx mori* are downloaded from [ftp://silkworm.swu.edu.cn](ftp://silkworm.swu.edu.cn/); *Daphnia pulex* from <http://wfleabase.org/>; *Pedicularis humanus* from <http://www.vectorbase.org/index.php>; *Drosophila melanogaster*, include gene annotation file in gff format, from [ftp.flybase.net](ftp://ftp.flybase.net/) release 5.8.

Bacteria (ftp://ftp.ncbi.nlm.nih.gov/genomes/Bacteria/all.fna.tar.gz 2009. 5).

Fungi (<ftp://ftp.ncbi.nlm.nih.gov/genomes/Fungi/>).

Rice (<ftp://ftp.ncbi.nlm.nih.gov/genomes/Oryza_sativa/*.ffn>).

Filtering transposable element and putative contamination sequences

We filtered transposable elements using RepeatProteinMasker [1] with e-value 0.001 based on Blastn. We also filter plant sequences (rice as a representative) using Blastn. For fungi, we select *Aspergillus fumigatus* from Ascomycetes class and *Magnaporthe grisea 70-15* from deuteromycetes class as representatives. All the Blastn e-values are set to 1e-10. Filter criteria: hit length >200 bp; and hit identity >80% for bacteria, >90% for fungi and plant.

Comparative Genomic Analysis

**Orthology.** All of the CDS and protein sequences are consistent with each other for every organism. Families and orthology were detected using previous methods [2,3], which involved two steps. 1) Assignment of pairwise relation. All-to-all protein sequence alignment is performed using Blastp of e-value < 1e-10. The fragmental alignments for each gene pairs are conjoined by solar. Then two genes are connected if the aligned region size ratio to both genes > 1/3. The similarity between two genes is weighted by an Hscore that rangs from 0 to 100. For two genes G1 and G2, the Hscore is defined as score(G1G2)/max( score(G1G1), score(G2G2) ). The score here is the BLAST raw score. 2) Extracting gene families. Genes are clustered by Hcluster_sg with parameters: the Hscore > 10, the minimum edge density (total number of edges / theoretical number of edges) > 1/3. The process of clustering a gene family would stop if the family has already included one or more genes from the outgroup.

**Phylogeny.** The phylogeny tree was reconstructed from 274 single-copy gene families, which were present in all species. The corresponding amino acid sequences were aligned with MUSCLE program and then trimmed to remove ambiguous sites or missing data. These trimmed alignments for each gene were then concatenated into a super-alignment containing 99, 128 positions [4].

The nucleotide and amino acid compositional bias (high A+T) of *Apis mellifera* can lead to the long-branch attraction artifact [5,6]. To address these problems, a Bayesian Markov chain Monte Carlo method, taking account of both process heterogeneity across lineages and process heterogeneity across sites, was implemented using CAT-BP model as described previously [7]. Chains were run for 4,000 cycles, each cycle resulting in a saved sample, and the first 2,400 samples were discarded as the ‘burn-in’. The analysis was repeated twice to approximate posteriors and check for convergence.

# Reference
