# Supplementary material for: De Novo Analysis of Transcriptome Dynamics in the Migratory Locust during the Development of Phase Traits
Source: PLoS One. 2010 Dec 30;5(12):e15633. doi: 10.1371/journal.pone.0015633 (PMC3012706; doi:10.1371/journal.pone.0015633)
Supplement: Figure S1 — Evaluation of assembled sequences. A. Assembled transcripts were compared with the evaluating EST set (11498 ESTs, with >90% coverage by at least 2X Illumina reads). B. Assembled transcripts were compared with full length cDNAs available in GenBank, and ORFs confirmed experimentally in our laboratory. C. An example of full length cDNA covered by assembled transcripts and ESTs. D. Five transcripts were validated by full length cDNA clones using RACE PCR. Y axis in the left (green bars) is the length of validated transcripts and y axis in the right (red points) is the ratio of validated regions. (DOC) [file pone.0015633.s002.doc]

**
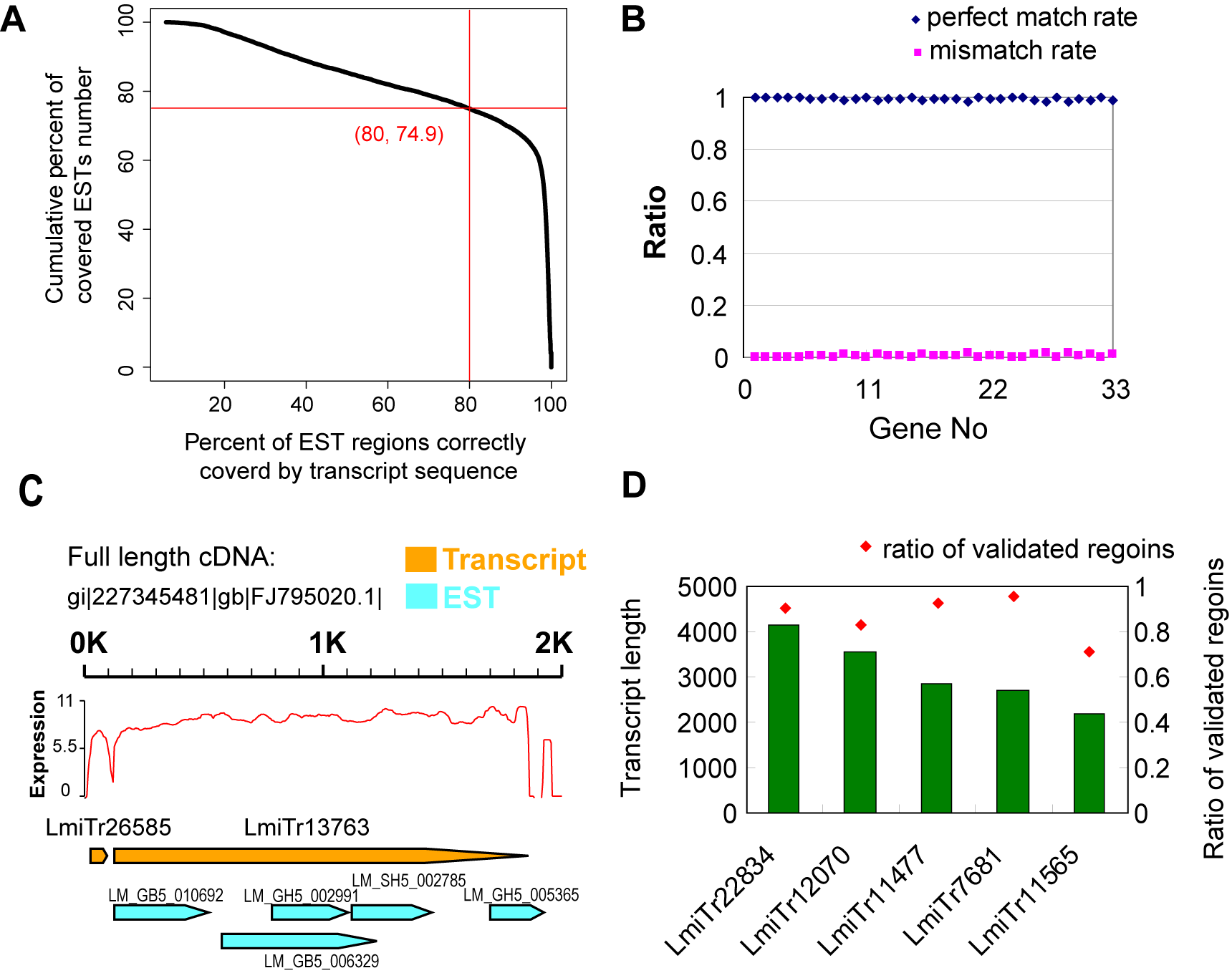
**

**Figure S1**

**Evaluation of assembled sequences.** A. Assembled transcripts were compared with the evaluating EST set (11498 ESTs, with > 90% coverage by at least 2X Illumina reads). B. Assembled transcripts were compared with full length cDNAs available in GenBank, and ORFs confirmed experimentally in our laboratory. C. An example of full length cDNA covered by assembled transcripts and ESTs. D. Five transcripts were validated by full length cDNA clones using RACE PCR. Y axis in the left (green bars) is the length of validated transcripts and y axis in the right (red points) is the ratio of validated regions.
