# Supplementary material for: De Novo Analysis of Transcriptome Dynamics in the Migratory Locust during the Development of Phase Traits
Source: PLoS One. 2010 Dec 30;5(12):e15633. doi: 10.1371/journal.pone.0015633 (PMC3012706; doi:10.1371/journal.pone.0015633)
Supplement: Figure S2 — Similarity search results against non-redundant (NR) database in NCBI. A. E-value distribution. B. Organism distribution. (DOC) [file pone.0015633.s003.doc]

**Figure S2**

**Similarity search results against non-redundant (NR) database in NCBI.** A. E value distribution. B. Organism distribution.
