# Supplementary material for: De Novo Analysis of Transcriptome Dynamics in the Migratory Locust during the Development of Phase Traits
Source: PLoS One. 2010 Dec 30;5(12):e15633. doi: 10.1371/journal.pone.0015633 (PMC3012706; doi:10.1371/journal.pone.0015633)
Supplement: Figure S3 — Real time PCR validation. * represents gene differential expression between two samples with significance. Real time PCR data were analyzed by independent two sample t test (P <0.05 for significance) and were represented by means ± SD. RNA-seq data of G4 and S4 were measured in RPKM. Transcripts for validation were selected randomly. Their annotations were listed according to the order in the figure as follows: VAT-1; Rho GTPase; Dorsal switch protein 1; ecdysone 20 hydroxylase; lipophorin receptor; Basement membrane-specific heparan sulfate proteoglycan core protein; TPR repeat-containing protein; chitinase; similar to CG32104; Mucin-5AC; taxilin alpha; conserved hypothetical protein. (DOC) [file pone.0015633.s004.doc]

**Figure S3**

**Real time PCR validation. *** represents gene differential expression between two samples with significance. Real time PCR data were analyzed by independent two sample t test (P < 0.05 for significance) and were represented by means ± SD. RNA-seq data of G4 and S4 were measured in RPKM. Transcripts for validation were selected randomly. Their annotations were listed according to the order in the figure as follows: VAT-1; Rho GTPase; Dorsal switch protein 1; ecdysone 20 hydroxylase; lipophorin receptor; Basement membrane-specific heparan sulfate proteoglycan core protein; TPR repeat-containing protein; chitinase; similar to CG32104; Mucin-5AC; taxilin alpha; conserved hypothetical protein.
