# Supplementary figures and images for: De Novo Analysis of Transcriptome Dynamics in the Migratory Locust during the Development of Phase Traits
Source: PLoS One. 2010 Dec 30;5(12):e15633. doi: 10.1371/journal.pone.0015633 (PMC3012706; doi:10.1371/journal.pone.0015633)

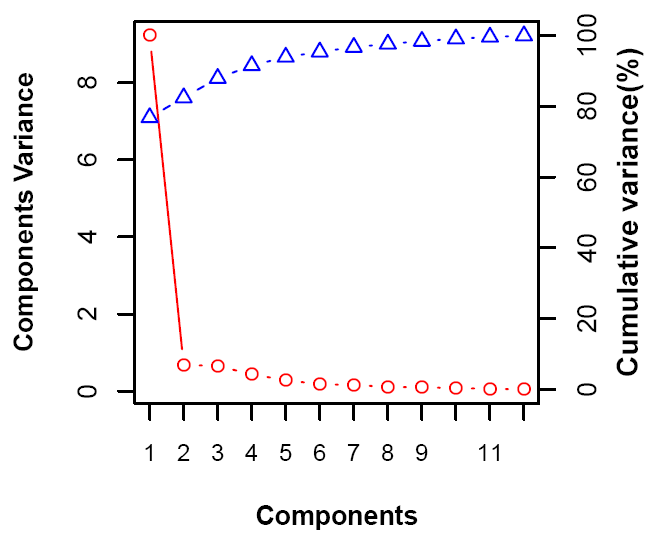


**Figure S4**

**Cumulative variance of PCs in principal component analysis of the 12 libraries.**

Supplement: Figure S4 — Cumulative variance of PCs in principal component analysis of the 12 libraries. (DOC) [file pone.0015633.s005.doc]
