# Supplementary material for: De Novo Analysis of Transcriptome Dynamics in the Migratory Locust during the Development of Phase Traits
Source: PLoS One. 2010 Dec 30;5(12):e15633. doi: 10.1371/journal.pone.0015633 (PMC3012706; doi:10.1371/journal.pone.0015633)
Supplement: Figure S5 — Embryonic and post-embryonic development of the locust. Left is the result of hierarchical clustering of differentially expressed transcripts (FDR<0.01, fold-change>2) generated by the pairwise comparison of all post-embryonic stages to the egg. It generated two main patterns. Red stands for up-regulating in post-embryonic stages (stages except the egg) and green stands for up-regulating in embryonic stage (the egg). Right is the KEGG enrichment of these two patterns. The Y axis is –log10 transformation of the p value calculated in enrichment test. Hierarchical clustering was performed using Gene Cluster 3.0. (DOC) [file pone.0015633.s006.doc]

**Figure S5**

**Embryonic and post-embryonic development of the locust.** Left is the result of hierarchical clustering of differentially expressed transcripts (FDR<0.01, fold-change>2) generated by the pairwise comparison of all post-embryonic stages to the egg. It generated two main patterns. Red stands for up-regulating in post-embryonic stages (stages except the egg) and green stands for up-regulating in embryonic stage (the egg). Right is the KEGG enrichment of these two patterns. The Y axis is –log_10_ transformation of the p value calculated in enrichment test. Hierarchical clustering was performed using Gene Cluster 3.0.
