# Supplementary material for: De Novo Analysis of Transcriptome Dynamics in the Migratory Locust during the Development of Phase Traits
Source: PLoS One. 2010 Dec 30;5(12):e15633. doi: 10.1371/journal.pone.0015633 (PMC3012706; doi:10.1371/journal.pone.0015633)
Supplement: Figure S6 — Adult and immature development of the locust. Left is the result of hierarchical clustering of differentially expressed transcripts (FDR<0.01, fold-change>2) generated by the pairwise comparison of all immature stages to the adult. It generated four main patterns. Red stands for up-regulating in immature stages (stages except the adult) and green stands for up-regulating in the mature stage (the adult). Right is the KEGG enrichment of these four patterns. The Y axis is –log10 transformation of the p value calculated in enrichment test. Hierarchical clustering was performed using Gene Cluster 3.0. (DOC) [file pone.0015633.s007.doc]

**Figure S6**

**Adult and immature development of the locust.** Left is the result of hierarchical clustering of differentially expressed transcripts (FDR<0.01, fold-change>2) generated by the pairwise comparison of all immature stages to the adult. It generated four main patterns. Red stands for up-regulation in immature stages (stages except the adult) and green stands for up-regulating in the mature stage (the adult). Right is the KEGG enrichment of these two patterns. The Y axis is –log_10_ transformation of the p value calculated in enrichment test. Hierarchical clustering was performed using Gene Cluster 3.0.
