# Supplementary material for: De Novo Analysis of Transcriptome Dynamics in the Migratory Locust during the Development of Phase Traits
Source: PLoS One. 2010 Dec 30;5(12):e15633. doi: 10.1371/journal.pone.0015633 (PMC3012706; doi:10.1371/journal.pone.0015633)
Supplement: Figure S7 — Number of differentially expressed transcripts (FDR<0.01, fold-change>2) in all development stages. (DOC) [file pone.0015633.s008.doc]

**Figure S7**

**Number of differentially expressed transcripts (FDR<0.01, fold-change>2) in all development stages.**
