# Supplementary material for: De Novo Analysis of Transcriptome Dynamics in the Migratory Locust during the Development of Phase Traits
Source: PLoS One. 2010 Dec 30;5(12):e15633. doi: 10.1371/journal.pone.0015633 (PMC3012706; doi:10.1371/journal.pone.0015633)
Supplement: Figure S8 — KEGG enrichment for gregarious up-regulated (red) or down regulated transcripts (blue) (FDR<10−5, fold-change>2) generated by pairwise comparison of the two deeply sequenced libraries (G4 and S4). The Y axis is –log10 transformation of the p value calculated in enrichment test. (DOC) [file pone.0015633.s009.doc]

**
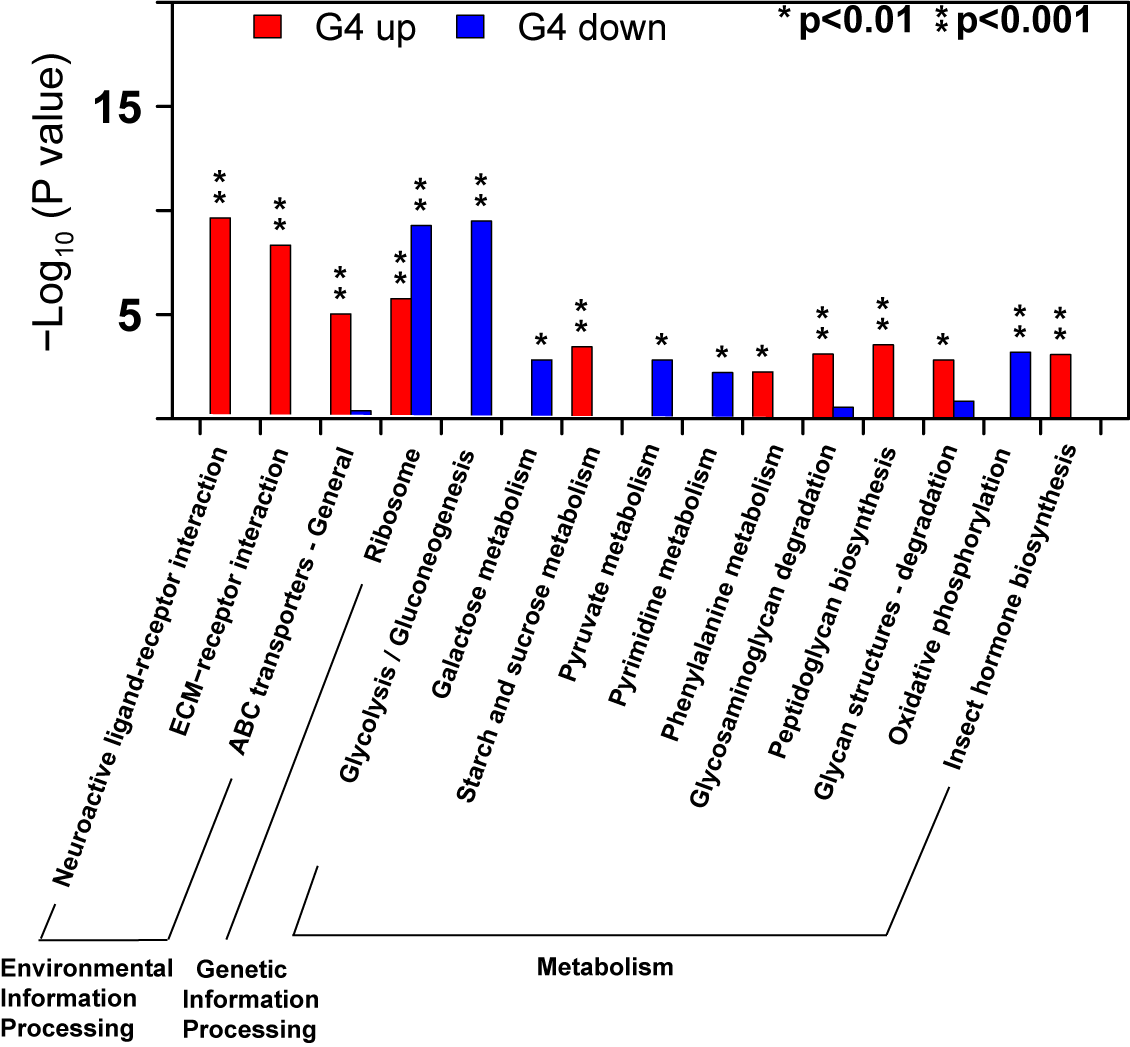
**

**Figure S8**

**KEGG enrichment for gregarious up-regulated (red) or down regulated transcripts (blue) (FDR<10^-5^, fold-change>2) generated by pairwise comparison of the two deeply sequenced libraries (G4 and S4).** The Y axis is –log_10_ transformation of the p value calculated in enrichment test.
