# Supplementary material for: De Novo Analysis of Transcriptome Dynamics in the Migratory Locust during the Development of Phase Traits
Source: PLoS One. 2010 Dec 30;5(12):e15633. doi: 10.1371/journal.pone.0015633 (PMC3012706; doi:10.1371/journal.pone.0015633)
Supplement: Figure S10 — A small functional network in locust transcriptome consisting of nuclear receptors and their interacting proteins. Red, green and gray colors denote being up- or down-regulated or showing no significance in G4 vs S4, respectively. Triangles represent transcription factors and spheres represent other proteins. (DOC) [file pone.0015633.s011.doc]

**
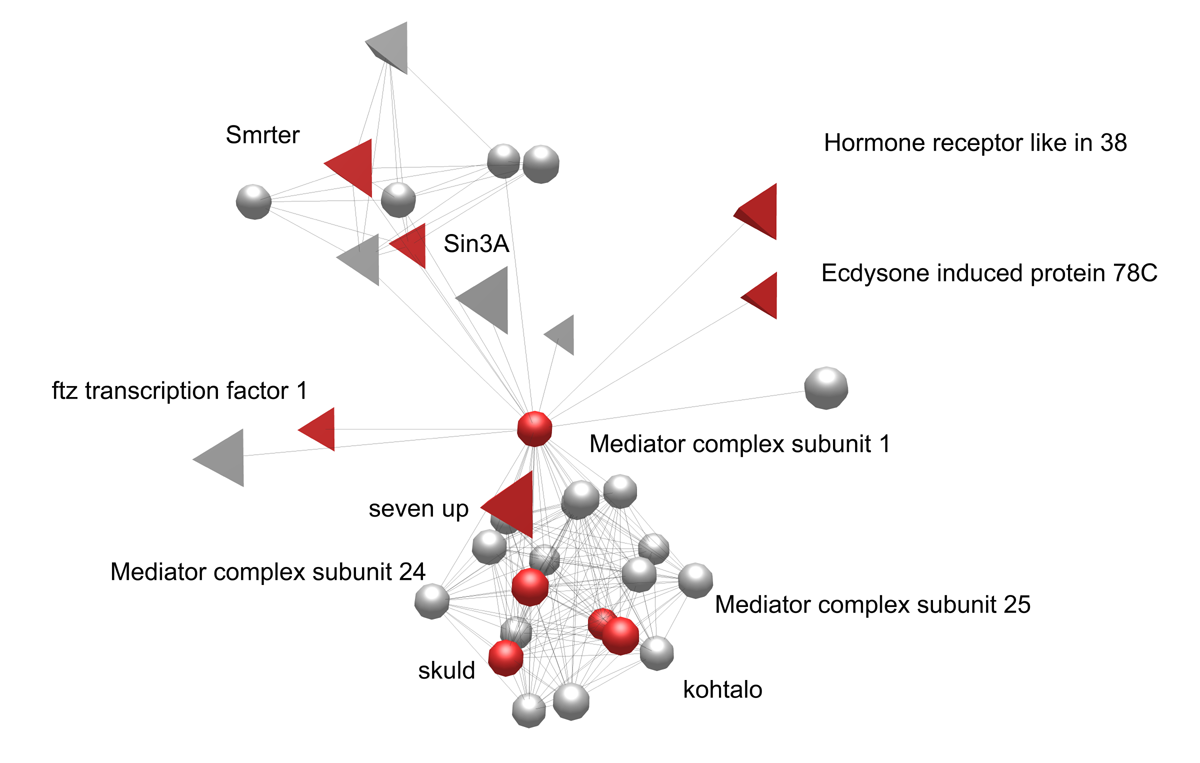
**

**Figure S10**

**A small functional network in locust transcriptome consisting of nuclear receptors and their interacting proteins.** Red, green and gray colors denote being up- or down-regulated or showing no significance in G4 vs S4, respectively. Triangles represent transcription factors and spheres represent other proteins.
