# Supplementary material for: De Novo Analysis of Transcriptome Dynamics in the Migratory Locust during the Development of Phase Traits
Source: PLoS One. 2010 Dec 30;5(12):e15633. doi: 10.1371/journal.pone.0015633 (PMC3012706; doi:10.1371/journal.pone.0015633)
Supplement: Figure S11 — Number of transcripts detected in different number of lanes in the deep sequencing data and length distribution of transcripts that can only be detected in lanes from G4 or S4. Sample G4 was sequenced in 16 lanes and S4 in 9 lanes. Reads of each lane from G4 and S4 that can be mapped to transcripts (that means this transcript can be detected in that lane) were recorded. Number of transcripts that can be detected using any N lanes from G4 or S4 was plotted. Group 1: N = 1, that means can be detected using any one lane in G4 or S4, i.e. expressing in all the lanes in G4 or S4; Group 2: N = 2∼8 for G4, N = 2∼4 for S4; Group 3: N = 9∼16 for G4, N = 5∼9 for S4. Red: can be detected only in lanes from G4; blue: can be detected only in lanes from S4; gray with strips: can be detected in lanes both from G4 and S4. On the top is the length distribution of the transcripts unique to one sample. (DOC) [file pone.0015633.s012.doc]

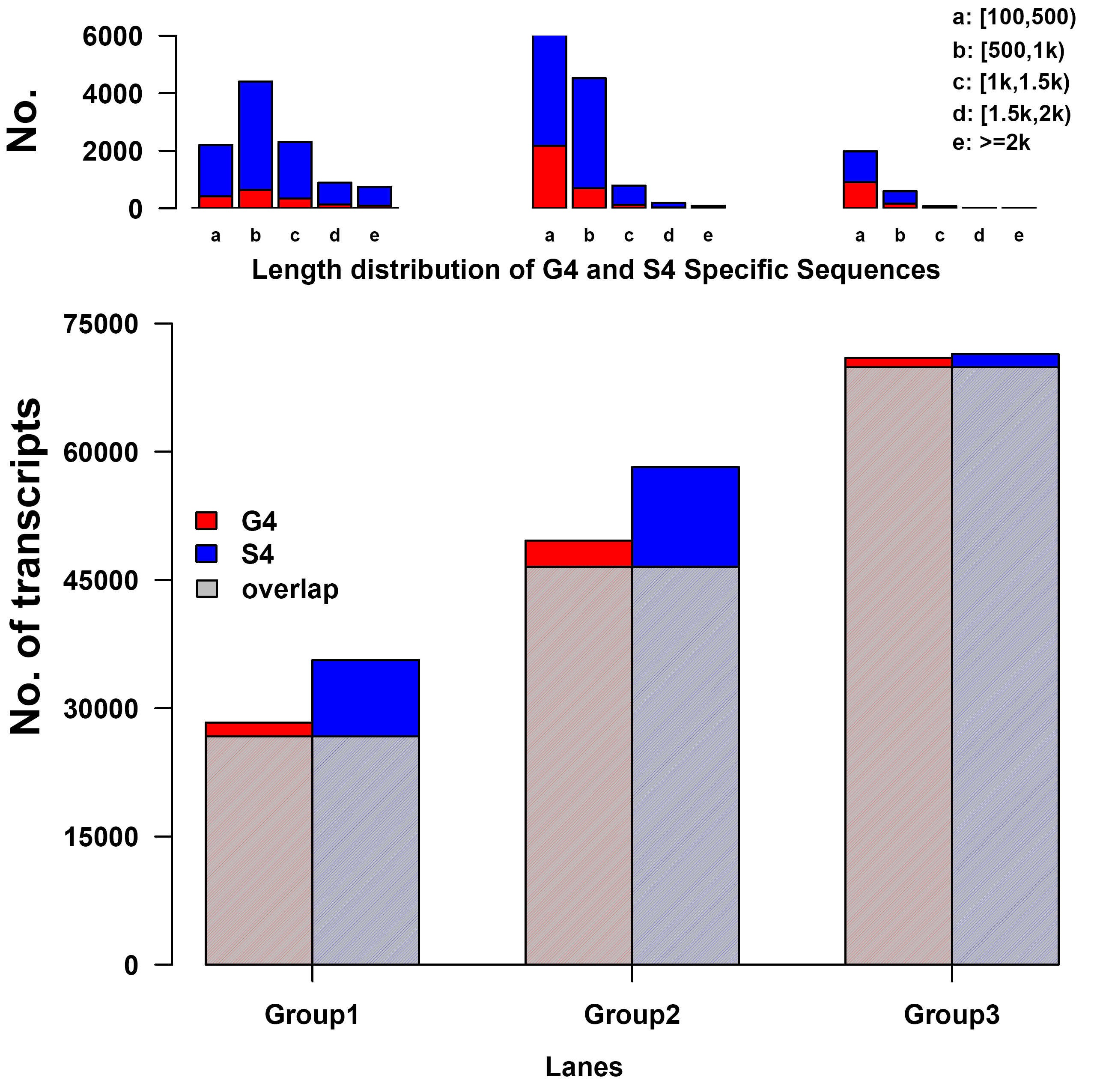


**Figure S11**

**Number of transcripts detected in different number of lanes in the deep sequencing data and length distribution of transcripts that can only be detected in lanes from G4 or S4.** Sample G4 was sequenced in 16 lanes and S4 in 9 lanes. Reads of each lane from G4 and S4 that can be mapped to transcripts (that means this transcript can be detected in that lane) were recorded. Number of transcripts that can be detected using any N lanes from G4 or S4 was plotted. Group 1: N=1, that means can be detected using any one lane in G4 or S4, i.e. expressing in all the lanes in G4 or S4; Group 2: N=2~8 for G4, N=2~4 for S4; Group 3: N=9~16 for G4, N=5~9 for S4. Red: can be detected only in lanes from G4; blue: can be detected only in lanes from S4; gray with strips: can be detected in lanes both from G4 and S4. On the top is the length distribution of the transcripts unique to one sample.
