# Supplementary material for: De Novo Analysis of Transcriptome Dynamics in the Migratory Locust during the Development of Phase Traits
Source: PLoS One. 2010 Dec 30;5(12):e15633. doi: 10.1371/journal.pone.0015633 (PMC3012706; doi:10.1371/journal.pone.0015633)
Supplement: Figure S12 — Length distribution of transcripts that express in different number of lanes. Sample G4 was sequenced in 16 lanes and S4 in 9 lanes. Reads of each lane from G4 and S4 that can be mapped to transcripts (that means this transcript expresses in that lane) were recorded. Every histogram represents the length distribution of transcripts that only express in N (the number in yellow strips) lanes in G4 (A) and S4 (B). For example, “1” in (A) represents transcripts that only express in one of the 16 lanes from G4, i.e. can only be detected by using all lanes; “16” in (A) represents transcripts that express in all the 16 lanes from G4, i.e. can be detected by using any one lane. X axis is the number of transcripts and Y axis is the length of transcripts (bp). (DOC) [file pone.0015633.s013.doc]

**Figure S12**

**Length distribution of transcripts that express in different number of lanes.** Sample G4 was sequenced in 16 lanes and S4 in 9 lanes. Reads of each lane from G4 and S4 that can be mapped to transcripts (that means this transcript expresses in that lane) were recorded. Every histogram represents the length distribution of transcripts that only express in N (the number in yellow strips) lanes in G4 (A) and S4 (B). For example, “1” in (A) represents transcripts that only express in one of the 16 lanes from G4, i.e. can only be detected by using all lanes; “16” in (A) represents transcripts that express in all the 16 lanes from G4, i.e. can be detected by using any one lane. X axis is the number of transcripts and Y axis is the length of transcripts (bp).
