# Supplementary material for: De Novo Analysis of Transcriptome Dynamics in the Migratory Locust during the Development of Phase Traits
Source: PLoS One. 2010 Dec 30;5(12):e15633. doi: 10.1371/journal.pone.0015633 (PMC3012706; doi:10.1371/journal.pone.0015633)
Supplement: Figure S13 — GO classification of the overlapping DETs and DETs specific to transcriptome or ESTs. WEGO was used to produce graphs. A. The second GO level. B. The third GO level of Cellular Component. (DOC) [file pone.0015633.s014.doc]

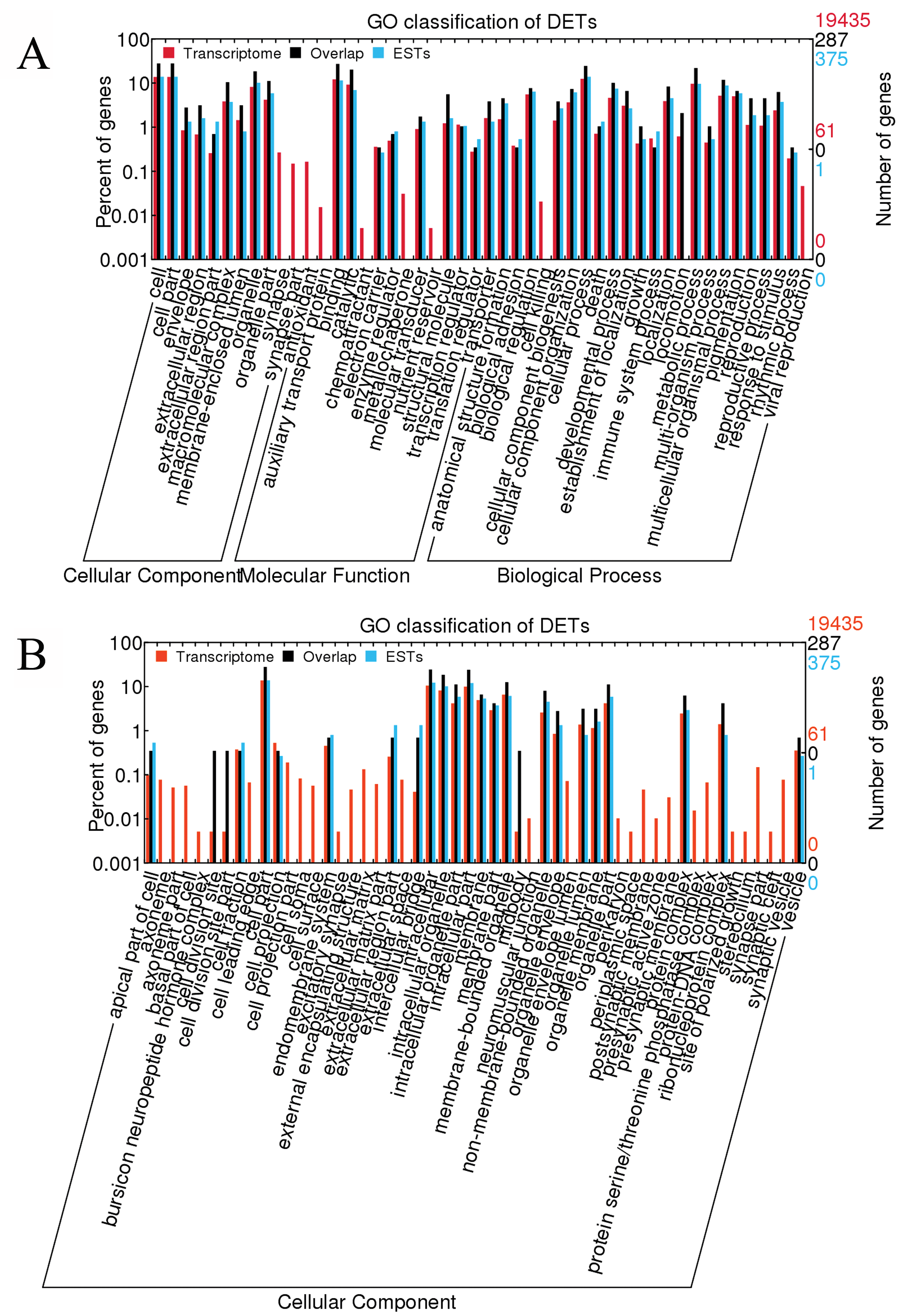


**Figure S13**

**GO classification of the overlapping DETs and DETs specific to transcriptome or ESTs.** WEGO was used to produce graphs. A. The second GO level. B. The third GO level of Cellular Component.
