# Supplementary material for: De Novo Analysis of Transcriptome Dynamics in the Migratory Locust during the Development of Phase Traits
Source: PLoS One. 2010 Dec 30;5(12):e15633. doi: 10.1371/journal.pone.0015633 (PMC3012706; doi:10.1371/journal.pone.0015633)
Supplement: Table S1 — Experimental design and sequence reads generated. (DOC) [file pone.0015633.s015.doc]

**Table S1. Experimental design and sequence reads generated**

| **Solitary samples** | **Total Reads** | **Total Length** | **Gregarious samples** | **Total Reads** | **Total Length** |
| --- | --- | --- | --- | --- | --- |
| **S egg** | 5,612,745 | 196,446,075 | **G egg** | 3,763,045 | 131,706,575 |
| **S 1+2** | 4,117,835 | 144,124,225 | **G 1+2** | 4,484,110 | 156,943,850 |
| **S 3** | 16,669,884 | 583,445,940 | **G 3** | 16,895,730 | 591,350,550 |
| **S 4** | 158,566,168 | 9,811,119,256 | **G 4** | 218,025,192 | 9,593,108,448 |
| **S 5** | 5,111,368 | 178,897,880 | **G 5** | 5,589,216 | 195,622,560 |
| **S adult** | 4,974,685 | 174,113,975 | **G adult** | 3,908,336 | 136,791,760 |
| **Total** | 195,052,835 | 10,733,446,251 | **Total** | 252,665,629 | 10,805,523,743 |
