# Supplementary material for: De Novo Analysis of Transcriptome Dynamics in the Migratory Locust during the Development of Phase Traits
Source: PLoS One. 2010 Dec 30;5(12):e15633. doi: 10.1371/journal.pone.0015633 (PMC3012706; doi:10.1371/journal.pone.0015633)
Supplement: Table S2 — Assembly statistics of contigs generated by assembly of the pool of all reads (PAAR) and assemblies of reads from G4 and S4 separately. (DOC) [file pone.0015633.s016.doc]

**Table S2. Assembly statistics of contigs** generated by assembly of the pool of all reads (PAAR) and assemblies of reads from G4 and S4 separately

| Contigs | K-mers 19 | | | K-mers 21 | | | K-mers 23 | | |
| --- | --- | --- | --- | --- | --- | --- | --- | --- | --- |
|  | PAAR | G4 | S4 | PAAR | G4 | S4 | PAAR | G4 | S4 |
| 1-50 | 19,321,477 | 5,466,442 | 10,308,334 | 12,931,084 | 3,511,555 | 7,414,019 | 9,425,830 | 2,418,931 | 5,614,862 |
| 50-100 | 383,526 | 238,767 | 217,388 | 284,485 | 169,737 | 178,695 | 253,793 | 129,165 | 166,906 |
| 100-150 | 104,717 | 63,533 | 67,258 | 84,145 | 51,461 | 60,383 | 79,603 | 48,212 | 60,909 |
| 150-200 | 43,495 | 28,570 | 31,975 | 34,718 | 21,483 | 28,972 | 31,939 | 19,737 | 28,270 |
| 200-500 | 52,677 | 43,849 | 45,270 | 50,243 | 33,933 | 43,415 | 46,345 | 29,716 | 42,526 |
| >=500 | 5,635 | 9,749 | 7,930 | 15,987 | 15,442 | 11,314 | 17,429 | 15,641 | 11,698 |
| Total NO. | 19,911,527 | 5,850,910 | 10,678,155 | 13,400,662 | 3,803,611 | 7,736,798 | 9,854,939 | 2,661,402 | 5,925,171 |
| Total Length (Mb) | 529.3 | 184.5 | 298.3 | 414.4 | 144.2 | 249.9 | 346.1 | 117.9 | 217.2 |
| Mean | 26.6 | 31.5 | 27.9 | 30.9 | 37.9 | 32.3 | 35.1 | 44.3 | 36.7 |
| Median | 21 | 22 | 21 | 24 | 25 | 24 | 26 | 27 | 26 |
| SD | 22.2 | 42.9 | 30.9 | 40.4 | 73.6 | 44.8 | 52.8 | 95.4 | 52.7 |
| Min | 20 | 20 | 20 | 22 | 22 | 22 | 24 | 24 | 24 |
| Max | 2,079 | 2,965 | 3,308 | 8,255 | 7,571 | 5,810 | 8,257 | 9,242 | 6,631 |
| N50 | 23 | 29 | 24 | 27 | 36 | 28 | 31 | 45 | 33 |
