# Supplementary material for: De Novo Analysis of Transcriptome Dynamics in the Migratory Locust during the Development of Phase Traits
Source: PLoS One. 2010 Dec 30;5(12):e15633. doi: 10.1371/journal.pone.0015633 (PMC3012706; doi:10.1371/journal.pone.0015633)
Supplement: Table S3 — Assembly statistics of scaffolds generated by assembly of the pool of all reads (PAAR) and assemblies of reads from G4 and S4 separately. (DOC) [file pone.0015633.s017.doc]

**Table S3. Assembly statistics of scaffolds generated by assembly of the pool of all reads (PAAR) and assemblies of reads from G4 and S4 separately**

| Scaffolds | K-mers 19 | | | K-mers 21 | | | K-mers 23 | | |
| --- | --- | --- | --- | --- | --- | --- | --- | --- | --- |
|  | PAAR | G4 | S4 | PAAR | G4 | S4 | PAAR | G4 | S4 |
| 100-500 | 90,573 | 44,362 | 62,886 | 73,905 | 31,983 | 53,201 | 64,140 | 26,606 | 50,550 |
| 500-1k | 17,663 | 10,142 | 18,808 | 19,509 | 9,931 | 18,422 | 18,413 | 8,884 | 18,048 |
| 1k-2k | 7,227 | 5,927 | 7,627 | 7,982 | 6,138 | 8,106 | 8,240 | 5,895 | 8,055 |
| >=2k | 4,237 | 4,900 | 3,559 | 5,119 | 5,366 | 4,287 | 5,600 | 5,479 | 4,569 |
| Total NO. | 119,700 | 65,331 | 92,880 | 106,515 | 53,418 | 84,016 | 96,393 | 46,864 | 81,222 |
| Total Length (Mb) | 54.2 | 43.8 | 46.0 | 58.5 | 43.9 | 47.7 | 58.4 | 42.1 | 47.8 |
| Mean | 453 | 670 | 495 | 549 | 821 | 567 | 606 | 898 | 588 |
| Median | 197 | 281 | 221 | 245 | 361 | 246 | 263 | 403 | 250 |
| SD | 766 | 1,118 | 716 | 928 | 1,299 | 836 | 1,032 | 1,374 | 866 |
| Min | 100 | 100 | 100 | 100 | 100 | 100 | 100 | 100 | 100 |
| Max | 18,191 | 24,455 | 16,177 | 20,158 | 25,461 | 22,009 | 25,666 | 22,875 | 20,167 |
| N50 | 869 | 1,559 | 919 | 1,069 | 1,873 | 1,086 | 1,254 | 2,061 | 1,138 |
