# Supplementary material for: De Novo Analysis of Transcriptome Dynamics in the Migratory Locust during the Development of Phase Traits
Source: PLoS One. 2010 Dec 30;5(12):e15633. doi: 10.1371/journal.pone.0015633 (PMC3012706; doi:10.1371/journal.pone.0015633)
Supplement: Table S4 — Transcriptome annotation by sequence similarity (e-value<1e-5). Sequences with length greater than 300 bp were subjected to annotation. InterPro was searched by InterProScan. GO was searched by Blast2GO. (DOC) [file pone.0015633.s018.doc]

**Table S4. Transcriptome annotation by sequence similarity (e-value<1e-5)**

**Sequences with length greater than 300bp were subjected to annotation. InterPro was searched by InterProScan. GO was searched by Blast2GO.**

| Database | Transcripts No. |
| --- | --- |
| Nr | 23,160 |
| Nt | 14,466 |
| SWISS-PROT | 19,767 |
| COG | 10,141 |
| KEGG | 14,973 |
| InterPro | 13,878 |
| GO | 10,475 |
| Total | 27,319 |
