# Supplementary material for: De Novo Analysis of Transcriptome Dynamics in the Migratory Locust during the Development of Phase Traits
Source: PLoS One. 2010 Dec 30;5(12):e15633. doi: 10.1371/journal.pone.0015633 (PMC3012706; doi:10.1371/journal.pone.0015633)
Supplement: Table S5 — Non-coding RNAs identified by BLASTN in locust transcriptome. The following non-coding databases were searched by BLASTN: RNAdb, NONCODE, Rfam, ncRNAdb and fRNAdb. Insect rRNAs were also searched. (DOC) [file pone.0015633.s019.doc]

**Table S5. Non-coding RNAs identified by BLASTN in locust transcriptome**

The following non-coding databases were searched by BLASTN: RNAdb, NONCODE, Rfam, ncRNAdb and fRNAdb. Insect rRNAs were also searched.

| locust id | Length (bp) | hit | Id in NONCODE or GenBank |
| --- | --- | --- | --- |
| LmiTr29792 | 122 | U6 | n4654_u375_M24607_snRNA |
| LmiTr65629 | 187 | U1 | n5337_u4688_X00549_snRNA |
| LmiTr14667 | 147 | 18S rRNA | gi|15986534|gb|AF370793.1| |
| LmiTr33315 | 1037 | 18S rRNA | gi|15986534|gb|AF370793.1| |
| LmiTr6299 | 2497 | 18S rRNA | gi|15986534|gb|AF370793.1| |
| LmiTr57568 | 183 | noncoding transcript | n197232_u193484_AK155563_mRNAlike |
