# Supplementary material for: De Novo Analysis of Transcriptome Dynamics in the Migratory Locust during the Development of Phase Traits
Source: PLoS One. 2010 Dec 30;5(12):e15633. doi: 10.1371/journal.pone.0015633 (PMC3012706; doi:10.1371/journal.pone.0015633)
Supplement: Table S6 — Hemimetabolous and holometabolous insect specific gene families. LMI: Locusta migratoria; PHU: Pedicularis humanus; API: Acyrthosiphon pisum; NVI: Nasonia vitripennis; AME: Apis mellifera; TCA: Tribolium castaneum; BMO: Bombyx mori; DME: Drosophila melanogaster; AGA: Anopheles gambiae. (DOC) [file pone.0015633.s020.doc]

**Table S6. Hemimetabolous and holometabolous insect specific gene families**

LMI*: Locusta migratoria;* PHU*: Pedicularis humanus;* API*: Acyrthosiphon pisum;* NVI: *Nasonia vitripennis*; AME*: Apis mellifera*; TCA: *Tribolium castaneum*; BMO: *Bombyx mori;* DME: *Drosophila melanogaster*; AGA: *Anopheles gambiae*.

| Family | Specific | Gene ID  Hemi ID: API  Holo ID: DME | LMI | PHU | API | AME | NVI | TCA | BMO | AGA | DME |
| --- | --- | --- | --- | --- | --- | --- | --- | --- | --- | --- | --- |
| 130 | Hemi | gi_193674121 | 1 | 1 | 1 | 0 | 0 | 0 | 0 | 0 | 0 |
| 150 |  | gi_193652624 | 1 | 1 | 1 | 0 | 0 | 0 | 0 | 0 | 0 |
| 960 |  | gi_193617689 | 1 | 1 | 1 | 0 | 0 | 0 | 0 | 0 | 0 |
| 972 |  | gi_193667020 | 1 | 1 | 1 | 0 | 0 | 0 | 0 | 0 | 0 |
| 2196 |  | gi_193711475 | 1 | 1 | 1 | 0 | 0 | 0 | 0 | 0 | 0 |
| 2303 |  | gi_193671793 | 1 | 1 | 1 | 0 | 0 | 0 | 0 | 0 | 0 |
| 2498 |  | gi_193610915 | 1 | 1 | 1 | 0 | 0 | 0 | 0 | 0 | 0 |
| 4320 |  | gi_193594179 | 1 | 1 | 1 | 0 | 0 | 0 | 0 | 0 | 0 |
| 4534 |  | gi_193643355 | 1 | 1 | 1 | 0 | 0 | 0 | 0 | 0 | 0 |
| 4854 |  | gi_193573494 | 1 | 1 | 1 | 0 | 0 | 0 | 0 | 0 | 0 |
| 4895 |  | gi_193697819 | 1 | 1 | 1 | 0 | 0 | 0 | 0 | 0 | 0 |
| 4909 |  | gi_193627509 | 1 | 1 | 1 | 0 | 0 | 0 | 0 | 0 | 0 |
| 5186 |  | gi_193699981 | 1 | 1 | 1 | 0 | 0 | 0 | 0 | 0 | 0 |
| 5593 |  | gi_193610586 | 1 | 1 | 1 | 0 | 0 | 0 | 0 | 0 | 0 |
| 5736 |  | gi_193697745 | 1 | 1 | 1 | 0 | 0 | 0 | 0 | 0 | 0 |
| 6020 |  | gi_193650171 | 1 | 1 | 1 | 0 | 0 | 0 | 0 | 0 | 0 |
| 6098 |  | gi_193587011 | 1 | 1 | 1 | 0 | 0 | 0 | 0 | 0 | 0 |
| 7438 |  | gi_193695260 | 1 | 1 | 1 | 0 | 0 | 0 | 0 | 0 | 0 |
| 8258 |  | gi_193659672 | 1 | 1 | 1 | 0 | 0 | 0 | 0 | 0 | 0 |
| 8495 |  | gi_193681027 | 1 | 1 | 1 | 0 | 0 | 0 | 0 | 0 | 0 |
| 8708 |  | gi_193678881 | 1 | 1 | 1 | 0 | 0 | 0 | 0 | 0 | 0 |
| 9311 |  | gi_193586937 | 1 | 1 | 1 | 0 | 0 | 0 | 0 | 0 | 0 |
| 9655 |  | gi_193695282 | 1 | 1 | 1 | 0 | 0 | 0 | 0 | 0 | 0 |
| 9854 |  | gi_193695150,gi_193575623 | 1 | 1 | 2 | 0 | 0 | 0 | 0 | 0 | 0 |
| 9857 |  | gi_193575629 | 1 | 1 | 1 | 0 | 0 | 0 | 0 | 0 | 0 |
| 9898 |  | gi_193580313 | 1 | 1 | 1 | 0 | 0 | 0 | 0 | 0 | 0 |
| 10129 |  | gi_193613118 | 1 | 1 | 1 | 0 | 0 | 0 | 0 | 0 | 0 |
| 10213 |  | gi_193631833 | 1 | 1 | 1 | 0 | 0 | 0 | 0 | 0 | 0 |
| 10231 |  | gi_193636613 | 1 | 1 | 1 | 0 | 0 | 0 | 0 | 0 | 0 |
| 10423 |  | gi_193690794 | 1 | 1 | 1 | 0 | 0 | 0 | 0 | 0 | 0 |
| 10441 |  | gi_193695278 | 1 | 1 | 1 | 0 | 0 | 0 | 0 | 0 | 0 |
| 10449 |  | gi_193697530 | 1 | 1 | 1 | 0 | 0 | 0 | 0 | 0 | 0 |
| 10474 |  | gi_193704624 | 1 | 1 | 1 | 0 | 0 | 0 | 0 | 0 | 0 |
| 10506 |  | gi_193716068 | 1 | 1 | 1 | 0 | 0 | 0 | 0 | 0 | 0 |
| 4 | Holo | FBpp0081535,FBpp0112313,FBpp0082844,FBpp0082843,FBpp0082857,FBpp0082845,FBpp0081536,FBpp0074135,FBpp0076886,FBpp0076963,FBpp0082847,FBpp0074095,FBpp0082846,FBpp0070282,FBpp0082859,FBpp0074136,FBpp0074138,FBpp0074137,FBpp0076964,FBpp0082858 | 0 | 0 | 0 | 3 | 28 | 7 | 2 | 19 | 20 |
| 540 |  | FBpp0076766,FBpp0076767,FBpp0076729,FBpp0076768,FBpp0076700,FBpp0076699,FBpp0076769,FBpp0076731,FBpp0076702,FBpp0111379,FBpp0076732,FBpp0076701,FBpp0076730,FBpp0076703 | 0 | 0 | 0 | 3 | 3 | 3 | 11 | 10 | 14 |
| 564 |  | FBpp0077623 | 0 | 0 | 0 | 4 | 2 | 1 | 1 | 1 | 1 |
| 879 |  | FBpp0070813 | 0 | 0 | 0 | 1 | 1 | 1 | 1 | 1 | 1 |
| 1756 |  | FBpp0081751 | 0 | 0 | 0 | 1 | 1 | 1 | 1 | 1 | 1 |
| 2538 |  | FBpp0112065 | 0 | 0 | 0 | 1 | 1 | 1 | 1 | 1 | 1 |
| 2662 |  | FBpp0112436 | 0 | 0 | 0 | 2 | 1 | 1 | 1 | 1 | 1 |
| 2899 |  | FBpp0075434 | 0 | 0 | 0 | 1 | 1 | 1 | 1 | 1 | 1 |
| 3207 |  | FBpp0081682 | 0 | 0 | 0 | 1 | 1 | 1 | 1 | 2 | 1 |
| 3627 |  | FBpp0072034 | 0 | 0 | 0 | 2 | 2 | 1 | 2 | 1 | 1 |
| 3703 |  | FBpp0084581 | 0 | 0 | 0 | 1 | 1 | 1 | 1 | 1 | 1 |
| 4346 |  | FBpp0086118,FBpp0080137 | 0 | 0 | 0 | 1 | 3 | 3 | 4 | 2 | 2 |
| 4488 |  | FBpp0082289,FBpp0074956,FBpp0074957,FBpp0086611,FBpp0082310,FBpp0074955 | 0 | 0 | 0 | 1 | 3 | 2 | 4 | 3 | 6 |
| 4820 |  | FBpp0074451,FBpp0074452,FBpp0074460 | 0 | 0 | 0 | 1 | 1 | 1 | 2 | 1 | 3 |
| 5515 |  | FBpp0086017,FBpp0086016 | 0 | 0 | 0 | 1 | 1 | 1 | 1 | 1 | 2 |
| 6885 |  | FBpp0078971 | 0 | 0 | 0 | 1 | 1 | 1 | 1 | 1 | 1 |
| 7427 |  | FBpp0081205 | 0 | 0 | 0 | 1 | 1 | 1 | 1 | 1 | 1 |
| 8169 |  | FBpp0084799 | 0 | 0 | 0 | 1 | 1 | 1 | 1 | 1 | 1 |
| 8675 |  | FBpp0080722 | 0 | 0 | 0 | 1 | 1 | 1 | 1 | 1 | 1 |
| 9745 |  | FBpp0084386 | 0 | 0 | 0 | 1 | 1 | 1 | 1 | 1 | 1 |
