# Supplementary material for: De Novo Analysis of Transcriptome Dynamics in the Migratory Locust during the Development of Phase Traits
Source: PLoS One. 2010 Dec 30;5(12):e15633. doi: 10.1371/journal.pone.0015633 (PMC3012706; doi:10.1371/journal.pone.0015633)
Supplement: Table S7 — Gene families underwent positive selection exclusively in hemimetabolous insects or in holometabolous insects. LMI: Locusta migratoria; PHU: Pedicularis humanus; API: Acyrthosiphon pisum; NVI: Nasonia vitripennis; AME: Apis mellifera; TCA: Tribolium castaneum; BMO: Bombyx mori; DME: Drosophila melanogaster; AGA: Anopheles gambiae. (DOC) [file pone.0015633.s021.doc]

**Table S7. Gene families underwent positive selection exclusively in hemimetabolous insects or in holometabolous insects.**

LMI*: Locusta migratoria;* PHU*: Pedicularis humanus;* API*: Acyrthosiphon pisum;* NVI: *Nasonia vitripennis*; AME*: Apis mellifera*; TCA: *Tribolium castaneum*; BMO: *Bombyx mori;* DME: *Drosophila melanogaster*; AGA: *Anopheles gambiae*.

| **Positive Selection** | | | **LMI, API, PHU** | | **TCA, BMO, AGA, DME, AME, NVI** | |
| --- | --- | --- | --- | --- | --- | --- |
|  | **FamilyID** | **Flybase ID** | **P value** | **Positive Sites** | **P value** | **Positive Sites** |
| Hemi positvie | 609 | FBpp0086706 | 1.65E-03 | yes | - | - |
|  | 954 | FBpp0074060 | 9.94E-04 | yes | - | - |
|  | 1398 | FBpp0073197 | 4.12E-05 | yes | - | - |
|  | 1913 | FBpp0099935 | 1.01E-02 | yes | - | - |
|  | 2490 | FBpp0073139 | 9.63E-03 | yes | - | - |
|  | 2584 | FBpp0076875 | 9.04E-04 | yes | - | - |
|  | 3300 | FBpp0081866 | 2.61E-03 | yes | - | - |
|  | 3550 | FBpp0271771 | 1.14E-03 | yes | - | - |
|  | 3955 | FBpp0111905 | 8.44E-07 | yes | - | - |
|  | 5843 | FBpp0271775 | 6.80E-05 | yes | - | - |
|  | 6364 | FBpp0079708 | 1.38E-02 | yes | - | - |
|  | 6974 | FBpp0077208 | 3.80E-03 | yes | - | - |
|  | 7542 | FBpp0074756 | 7.85E-03 | yes | - | - |
|  | 7713 | FBpp0089346 | 2.62E-03 | yes | - | - |
|  | 7715 | FBpp0288673 | 1.74E-02 | yes | - | - |
|  | 8019 | FBpp0078941 | 1.01E-02 | yes | - | - |
|  | 9102 | FBpp0075727 | 1.08E-02 | yes | - | - |
|  | 9261 | FBpp0084940 | 6.66E-04 | yes | - | - |
|  | 9289 | FBpp0071993 | 4.31E-03 | yes | - | - |
|  | 9452 | FBpp0078980 | 7.51E-03 | yes | - | - |
|  | 9759 | FBpp0070931 | 1.02E-03 | yes | - | - |
| Holo positve | 222 | FBpp0072874 | - | - | 1.05E-02 | yes |
|  | 237 | FBpp0076337 | - | - | 9.62E-04 | yes |
|  | 410 | FBpp0074936 | - | - | 8.97E-05 | yes |
|  | 1157 | FBpp0072450 | - | - | 7.57E-07 | yes |
|  | 1331 | FBpp0077147 | - | - | 1.07E-03 | yes |
|  | 1768 | FBpp0072366 | - | - | 2.37E-03 | yes |
|  | 1778 | FBpp0079812 | - | - | 1.81E-02 | yes |
|  | 1799 | FBpp0080319 | - | - | 8.18E-03 | yes |
|  | 2086 | FBpp0081262 | - | - | 1.06E-03 | yes |
|  | 2388 | FBpp0085571 | - | - | 9.82E-04 | yes |
|  | 2389 | FBpp0072602 | - | - | 9.91E-03 | yes |
|  | 2409 | FBpp0075212 | - | - | 2.12E-03 | yes |
|  | 2528 | FBpp0071516 | - | - | 2.09E-04 | yes |
|  | 2600 | FBpp0078636 | - | - | 1.74E-02 | yes |
|  | 2682 | FBpp0080630 | - | - | 1.88E-04 | yes |
|  | 2798 | FBpp0074650 | - | - | 2.57E-04 | yes |
|  | 3023 | FBpp0110267 | - | - | 1.90E-03 | yes |
|  | 3312 | FBpp0074529 | - | - | 4.30E-04 | yes |
|  | 3963 | FBpp0086011 | - | - | 1.34E-02 | yes |
|  | 4263 | FBpp0075784 | - | - | 3.13E-06 | yes |
|  | 4393 | FBpp0082065 | - | - | 1.49E-02 | yes |
|  | 4638 | FBpp0085773 | - | - | 9.52E-03 | yes |
|  | 4639 | FBpp0085959 | - | - | 5.16E-03 | yes |
|  | 5615 | FBpp0087733 | - | - | 3.64E-04 | yes |
|  | 5782 | FBpp0083894 | - | - | 1.02E-04 | yes |
|  | 5909 | FBpp0080024 | - | - | 5.08E-04 | yes |
|  | 5932 | FBpp0087353 | - | - | 8.90E-07 | yes |
|  | 6157 | FBpp0077714 | - | - | 7.42E-04 | yes |
|  | 6181 | FBpp0072672 | - | - | 6.25E-06 | yes |
|  | 6187 | FBpp0077650 | - | - | 1.78E-03 | yes |
|  | 6743 | FBpp0110281 | - | - | 8.86E-03 | yes |
|  | 7236 | FBpp0087755 | - | - | 4.55E-07 | yes |
|  | 7237 | FBpp0072788 | - | - | 3.32E-03 | yes |
|  | 7601 | FBpp0082984 | - | - | 1.10E-04 | yes |
|  | 7967 | FBpp0079951 | - | - | 5.34E-04 | yes |
|  | 8151 | FBpp0079914 | - | - | 6.73E-03 | yes |
|  | 8368 | FBpp0070295 | - | - | 2.64E-03 | yes |
|  | 8469 | FBpp0083166 | - | - | 9.44E-04 | yes |
|  | 8546 | FBpp0083658 | - | - | 7.71E-05 | yes |
|  | 8621 | FBpp0087760 | - | - | 1.19E-03 | yes |
|  | 8895 | FBpp0082028 | - | - | 1.56E-02 | yes |
|  | 8928 | FBpp0083264 | - | - | 1.36E-05 | yes |
|  | 8976 | FBpp0076818 | - | - | 2.51E-03 | yes |
|  | 9000 | FBpp0085801 | - | - | 6.79E-03 | yes |
|  | 9082 | FBpp0075238 | - | - | 2.09E-03 | yes |
|  | 9087 | FBpp0072801 | - | - | 9.16E-03 | yes |
|  | 9135 | FBpp0073354 | - | - | 5.27E-03 | yes |
|  | 9167 | FBpp0079454 | - | - | 4.69E-08 | yes |
|  | 9329 | FBpp0088527 | - | - | 6.79E-05 | yes |
|  | 9397 | FBpp0079607 | - | - | 4.32E-03 | yes |
|  | 9418 | FBpp0083159 | - | - | 6.32E-03 | yes |
|  | 9449 | FBpp0071045 | - | - | 5.80E-04 | yes |
|  | 9555 | FBpp0074709 | - | - | 1.67E-03 | yes |
|  | 9746 | FBpp0071808 | - | - | 1.58E-02 | yes |
