# Supplementary material for: De Novo Analysis of Transcriptome Dynamics in the Migratory Locust during the Development of Phase Traits
Source: PLoS One. 2010 Dec 30;5(12):e15633. doi: 10.1371/journal.pone.0015633 (PMC3012706; doi:10.1371/journal.pone.0015633)
Supplement: Table S8 — Gene families under adaptation pressures which affect development process. (DOC) [file pone.0015633.s022.doc]

**Table S8. Gene families under adaptation pressures which affect development process**

|  | Family ID | Development Relevant Functions |
| --- | --- | --- |
| Hemi specific | 972 | anti apoptosis |
|  | 8708 | cell morphogenesis |
|  | 10213 | DNA replication, organ morphogenesis |
| Holo specific | 540 | development, chitin-based larval cuticle |
|  | 879 | germ cell programmed cell death |
|  | 4488 | hemocyte differentiation, spermatid development |
|  | 4820 | germ cell programmed cell death |
|  | 8675 | development,eye photoreceptor cell differentiation |
| Hemi positive | 954 | mushroom body development, compound eye development, activation of MAPK activity |
|  | 3550 | instar larval development, open tracheal system development |
|  | 6974 | oocyte development |
|  | 8019 | muscle development |
| Holo positive | 1331 | induction of compound eye retinal cell programmed cell death |
|  | 2388 | mushroom body development |
|  | 3023 | nervous system development |
|  | 3963 | a regulator of dpp signal pathway in embryonic dorsal-ventral patterning |
|  | 4263 | embryonic development via the syncytial blastoderm, axon guidance, brain morphogenesis |
|  | 6181 | instar larval development |
