# Supplementary material for: De Novo Analysis of Transcriptome Dynamics in the Migratory Locust during the Development of Phase Traits
Source: PLoS One. 2010 Dec 30;5(12):e15633. doi: 10.1371/journal.pone.0015633 (PMC3012706; doi:10.1371/journal.pone.0015633)
Supplement: Table S9 — Distribution of differentially expressed transcripts (DETs) in PC1. (DOC) [file pone.0015633.s023.doc]

**Table S9. Distribution of differentially expressed transcripts (DETs) in PC1**

| PC1 score | DETs No. | Percentage |
| --- | --- | --- |
| 2 < score ≤ 5 | 166 | 1.46% |
| 1 < score ≤ 2 | 539 | 9.99% |
| 0 <s core ≤ 1 | 774 | 18.05% |
| -1 < score ≤ 0 | 956 | 24.54% |
| -2 < score ≤ -1 | 1060 | 33.54% |
| -3 < score ≤ -2 | 942 | 42.53% |
| -4 < score ≤ -3 | 871 | 55.27% |
| -5 < score ≤ -4 | 723 | 67.70% |
| -10 < score ≤ -5 | 1579 | 78.64% |
| -25 < score ≤ -10 | 371 | 84.70% |
