# Supplementary material for: De Novo Analysis of Transcriptome Dynamics in the Migratory Locust during the Development of Phase Traits
Source: PLoS One. 2010 Dec 30;5(12):e15633. doi: 10.1371/journal.pone.0015633 (PMC3012706; doi:10.1371/journal.pone.0015633)
Supplement: Table S10 — The index number in Figure 4A and the enriched (p<0.01) GO categories of gregarious up-regulated or down regulated transcripts (FDR<0.01, fold-change>2) generated by pairwise comparison of the two phases in egg, 1st and 2nd instar, the 3rd instar, 4th instar, 5th instar, and adult. (DOC) [file pone.0015633.s024.doc]

**Table S10. The index number in Figure 3A and the enriched (p<0.01) GO categories of gregarious up-regulated or down regulated transcripts (FDR<0.01, fold-change>2) generated by pairwise comparison of the two phases in egg, 1st and 2nd instar, the 3rd instar, 4th instar, 5th-instar, and adult**

| Functional Category  (the second GO level) | Index Number in Figure 3 | Enriched GO Category  (the third GO level) |
| --- | --- | --- |
| Metabolic process | 1 | nitrogen compound metabolic process |
|  | 2 | catabolic process |
|  | 3 | biosynthetic process |
|  | 4 | secondary metabolic process |
|  | 5 | macromolecule metabolic process |
|  | 6 | cellular metabolic process |
|  | 7 | primary metabolic process |
|  | 8 | oxidation reduction |
| Cellular process | 9 | cell cycle |
|  | 10 | cell cycle process |
|  | 11 | cytokinetic process |
| Catalytic activity | 12 | isomerase activity |
|  | 13 | RNA splicing factor activity, transesterification mechanism |
|  | 14 | oxidoreductase activity |
|  | 15 | hydrolase activity |
|  | 16 | lyase activity |
| Organelle | 17 | membrane-bounded organelle |
|  | 18 | non-membrane-bounded organelle |
|  | 19 | intracellular organelle |
|  | 20 | organelle part |
| Organelle part | 21 | organelle membrane |
|  | 22 | intracellular organelle part |
| Membrane-enclosed lumen | 23 | organelle envelope lumen |
|  | 24 | organelle lumen |
| Cell part | 25 | intracellular |
|  | 26 | intracellular part |
|  | 27 | endomembrane system |
|  | 28 | periplasmic space |
|  | 29 | external encapsulating structure |
| Macromolecular complex | 30 | ribonucleoprotein complex |
|  | 31 | protein complex |
| Binding | 32 | drug binding |
|  | 33 | lipid binding |
|  | 34 | nucleic acid binding |
|  | 35 | pattern binding |
|  | 36 | carbohydrate binding |
|  | 37 | tetrapyrrole binding |
| Multicellular organismal process | 38 | respiratory gaseous exchange |
|  | 39 | adult behavior |
|  | 40 | molting cycle |
| Structural molecule activity | 41 | structural constituent of muscle |
|  | 42 | structural constituent of cuticle |
|  | 43 | structural constituent of ribosome |
| Other | 44 | response to other organism |
|  | 45 | detection of stimulus |
|  | 46 | mating |
|  | 47 | positive regulation of anti-apoptosis |
|  | 48 | pseudocleavage |
|  | 49 | substrate-specific transporter activity |
|  | 50 | translation factor activity, nucleic acid binding |
|  | 51 | enzyme inhibitor activity |
|  | 52 | copper chaperone activity |
|  | 53 | establishment of protein localization |
|  | 54 | peroxidase activity |
|  | 55 | extracellular matrix |
|  | 56 | organelle envelope |
|  | 57 | cell part |
