# Supplementary material for: De Novo Analysis of Transcriptome Dynamics in the Migratory Locust during the Development of Phase Traits
Source: PLoS One. 2010 Dec 30;5(12):e15633. doi: 10.1371/journal.pone.0015633 (PMC3012706; doi:10.1371/journal.pone.0015633)
Supplement: Table S11 — The index number in Figure 4B and the enriched (p<0.01) KEGG pathways of gregarious up-regulated or down regulated transcripts (FDR<0.01, fold-change>2) generated by pairwise comparison of the two phases in egg, 1st and 2nd instar, the 3rd instar, 4th instar, 5th instar, and adult. (DOC) [file pone.0015633.s025.doc]

**Table S11. The index number in Figure 3B and the enriched (p<0.01) KEGG pathways of gregarious up-regulated or down regulated transcripts (FDR<0.01, fold-change>2) generated by pairwise comparison of the two phases in egg, 1st and 2nd instar, the 3rd instar, 4th instar, 5th-instar, and adult**

| Functional Category | | Index Number in Figure 4 | Enriched KEGG Category |
| --- | --- | --- | --- |
| Environmental Information Processing | Membrane Transport | 1 | ABC transporters - General |
|  | Signaling Molecules and Interaction | 2 | Neuroactive ligand-receptor interaction |
|  |  | 3 | ECM-receptor interaction |
| Metabolism | Amino Acid Metabolism | 4 | Phenylalanine, tyrosine and tryptophan biosynthesis |
|  |  | 5 | Glycine, serine and threonine metabolism |
|  |  | 6 | Alanine and aspartate metabolism |
|  |  | 7 | Phenylalanine metabolism |
|  |  | 8 | Valine, leucine and isoleucine degradation |
|  |  | 9 | Lysine degradation |
|  | Carbohydrate Metabolism | 10 | Starch and sucrose metabolism |
|  |  | 11 | Glyoxylate and dicarboxylate metabolism |
|  |  | 12 | Pentose phosphate pathway |
|  |  | 13 | Ascorbate and aldarate metabolism |
|  |  | 14 | Galactose metabolism |
|  |  | 15 | Pentose and glucuronate interconversions |
|  |  | 16 | Propanoate metabolism |
|  |  | 17 | Butanoate metabolism |
|  |  | 18 | Glycolysis / Gluconeogenesis |
|  |  | 19 | Citrate cycle (TCA cycle) |
|  | Glycan Biosynthesis and Metabolism | 20 | Glycosphingolipid biosynthesis - ganglio series |
|  |  | 21 | N-Glycan degradation |
|  |  | 22 | Glycan structures - degradation |
|  |  | 23 | Glycosaminoglycan degradation |
|  | Lipid Metabolism | 24 | Sphingolipid metabolism |
|  |  | 25 | Glycerolipid metabolism |
|  |  | 26 | Fatty acid metabolism |
|  |  | 27 | Fatty acid elongation in mitochondria |
|  |  | 28 | Bile acid biosynthesis |
|  |  | 29 | Arachidonic acid metabolism |
|  | Metabolism of Other Amino Acids | 30 | Glutathione metabolism |
|  |  | 31 | beta-Alanine metabolism |
|  |  | 32 | Selenoamino acid metabolism |
|  | Energy Metabolism | 33 | Oxidative phosphorylation |
|  | Metabolism of Cofactors and Vitamins | 34 | One carbon pool by folate |
|  |  | 35 | Porphyrin and chlorophyll metabolism |
|  |  | 36 | Folate biosynthesis |
|  | Metabolism of Terpenoids and Polyketides | 37 | Insect hormone biosynthesis |
|  | Nucleotide Metabolism | 38 | Pyrimidine metabolism |
|  | Xenobiotics Biodegradation and Metabolism | 39 | Metabolism of xenobiotics by cytochrome P450 |
| Genetic Information Processing | Folding, Sorting and Degradation | 40 | SNARE interactions in vesicular transport |
|  |  | 41 | Proteasome |
|  | Replication and Repair | 42 | DNA replication |
|  | Transcription | 43 | RNA polymerase |
|  | Translation | 44 | Ribosome |
| Cellular Processes | Cell Communication | 45 | Focal adhesion |
|  |  | 46 | Adherens junction |
|  | Cell Motility | 47 | Regulation of actin cytoskeleton |
| Organismal Systems | Development | 48 | Dorso-ventral axis formation |
|  | Environmental Adaptation | 49 | Circadian rhythm |
