# Supplementary material for: De Novo Analysis of Transcriptome Dynamics in the Migratory Locust during the Development of Phase Traits
Source: PLoS One. 2010 Dec 30;5(12):e15633. doi: 10.1371/journal.pone.0015633 (PMC3012706; doi:10.1371/journal.pone.0015633)
Supplement: Table S12 — Candidates for phase marker genes. Candidates with clear annotations were listed. (DOC) [file pone.0015633.s026.doc]

**Table S12. Candidates for phase marker genes**

Candidates with clear annotations were listed.

| Marker | | ID | Annotation |
| --- | --- | --- | --- |
| S | Specific | LmiTr10242 | Blue (type 1) copper domain |
|  |  | LmiTr10742 | Envelope glycoprotein |
|  |  | LmiTr14216 | similar to forkhead box transcription factor subgroup N1 |
|  |  | LmiTr16555 | Zinc finger |
|  |  | LmiTr19871 | toll, putative |
|  |  | LmiTr19949 | Immunoglobulin/major histocompatibility complex, conserved site |
|  |  | LmiTr20893 | structural constituent of cuticle |
|  |  | LmiTr22762 | similar to AGAP006594-PA |
|  |  | LmiTr23730 | IPR001611; Leucine-rich repeat |
|  |  | LmiTr25566 | similar to AGAP003204-PA |
|  |  | LmiTr26010 | similar to phospholipase c epsilon |
|  |  | LmiTr27012 | Down syndrome cell adhesion molecule isoform |
|  |  | LmiTr2907 | ATPase, F1/V1/A1 complex, alpha/beta subunit, nucleotide-binding |
|  |  | LmiTr33603 | similar to p20-CGGBP |
|  |  | LmiTr35252 | mitochondrial ornithine transporter |
|  |  | LmiTr35336 | similar to Zinc carboxypeptidase family protein |
|  |  | LmiTr36044 | choline/ethanolamine kinase |
|  |  | LmiTr3651 | arylsulfatase |
|  |  | LmiTr36599 | Cytochrome P450 |
|  |  | LmiTr37427 | translation initiation factor 5a |
|  |  | LmiTr38792 | similar to Egg-derived tyrosine phosphatase CG6542-PA |
|  |  | LmiTr38857 | Peptidoglycan-binding domain 1 protein |
|  |  | LmiTr40231 | similar to pol polyprotein |
|  |  | LmiTr41045 | similar to AGAP003417-PA |
|  |  | LmiTr41265 | similar to combgap |
|  |  | LmiTr42914 | similar to gag-pol polyprotein |
|  |  | LmiTr43323 | transposase |
|  |  | LmiTr43728 | tyrosine kinase receptor |
|  |  | LmiTr44523 | similar to CG8785-PA |
|  |  | LmiTr44759 | insulin receptor tyrosine kinase substrate |
|  |  | LmiTr45530 | similar to ENSANGP00000019247 |
|  |  | LmiTr45591 | similar to gag-pol polyprotein |
|  |  | LmiTr46089 | PREDICTED: similar to CG1213-PA |
|  |  | LmiTr46392 | Ankyrin |
|  |  | LmiTr47322 | homeobox protein prospero/prox-1 |
|  |  | LmiTr48318 | sugar transporter |
|  |  | LmiTr48334 | similar to AGAP005245-PD isoform 4 |
|  |  | LmiTr48569 | Na[+]-dependent inorganic phosphate cotransporter |
|  |  | LmiTr49004 | similar to echinus CG2904-PB |
|  |  | LmiTr49017 | CLIP-domain serine protease subfamily B |
|  |  | LmiTr49045 | Hairy protein |
|  |  | LmiTr49976 | similar to ENSANGP00000024305 |
|  |  | LmiTr50705 | similar to Insulin receptor tyrosine kinase substrate |
|  |  | LmiTr51485 | similar to CG6330 |
|  |  | LmiTr51519 | fructose 1,6-bisphosphate aldolase |
|  |  | LmiTr52010 | geminin |
|  |  | LmiTr52235 | DEATH-like |
|  |  | LmiTr52586 | Acyl-CoA synthetase family member |
|  |  | LmiTr53319 | Zinc finger |
|  |  | LmiTr53349 | similar to APC-like CG1451-PA |
|  |  | LmiTr53582 | mucin 5, subtype B, tracheobronchial, partial |
|  |  | LmiTr54690 | similar to polyprotein |
|  |  | LmiTr54697 | similar to CG12071-PB |
|  |  | LmiTr70690 | similar to GF15004 |
|  |  | LmiTr7650 | ATPase, F0 complex, subunit C |
|  |  | LmiTr8957 | similar to spalt major |
|  |  | LmiTr9307 | reverse transcriptase |
|  |  | LmiTr9570 | RNA-directed RNA polymerase catalytic subunit |
|  |  | LmiTr11181 | similar to amontillado CG6438-PA |
|  | Stable Difference | LmiTr37778 | similar to klarsicht CG17046-PA |
|  |  | LmiTr13236 | neurotactin |
|  |  | LmiTr33431 | similar to Choline/ethanolamine kinase |
|  |  | LmiTr18413 | similar to Choline/ethanolamine kinase |
|  |  | LmiTr8056 | similar to Darkener of apricot CG33553-PG |
|  |  | LmiTr52217 | Dentin sialophosphoprotein precursor |
|  |  | LmiTr15416 | similar to GF11368 |
|  |  | LmiTr3607 | similar to CG31132-PA |
| G | Specific | LmiTr11466 | similar to activating transcription factor 2 isoform 1 |
|  |  | LmiTr11574 | membrane-associated RING finger protein |
|  |  | LmiTr1234 | mfs transporter |
|  |  | LmiTr12896 | carbohydrate kinase-like |
|  |  | LmiTr1338 | cytoplasmic dynein heavy chain |
|  |  | LmiTr14105 | defensin |
|  |  | LmiTr14631 | Chain A, Ketose Reductase |
|  |  | LmiTr15638 | ankyrin 2,3/unc44 |
|  |  | LmiTr18706 | cadherin-like Cry1Ac receptor |
|  |  | LmiTr18848 | similar to CG7627-PA |
|  |  | LmiTr19471 | Pyridoxal phosphate phosphatase |
|  |  | LmiTr19556 | ubiquitin-conjugating enzyme rad6 |
|  |  | LmiTr1972 | similar to 120.7 kDa protein in NOF-FB transposable element |
|  |  | LmiTr19894 | galactosidase, beta 1-like 2 |
|  |  | LmiTr20262 | Glycine N-methyltransferase |
|  |  | LmiTr20281 | guanylate cyclase |
|  |  | LmiTr20359 | gag-pol polyprotein |
|  |  | LmiTr20678 | UNC93A protein |
|  |  | LmiTr20712 | N-acetylgalactosaminyltransferase |
|  |  | LmiTr21541 | Cystinosin homolog |
|  |  | LmiTr21652 | similar to CG9084 |
|  |  | LmiTr22176 | ankyrin 2,3/unc44 |
|  |  | LmiTr22448 | similar to AGAP004232-PA |
|  |  | LmiTr22643 | nuclear receptor |
|  |  | LmiTr22835 | similar to rotatin |
|  |  | LmiTr23246 | Phosphotransferase system, HPr histidine phosphorylation site |
|  |  | LmiTr23484 | zinc finger protein, putative |
|  |  | LmiTr24169 | Guanylyl cyclase at 89Db CG14886-PA |
|  |  | LmiTr24270 | phospholipase |
|  |  | LmiTr2473 | galactosidase |
|  |  | LmiTr25060 | endonuclease and reverse transcriptase-like protein |
|  |  | LmiTr25802 | similar to cpg binding protein |
|  |  | LmiTr26059 | similar to GM26411 |
|  |  | LmiTr26148 | Uncharacterized protein U88 |
|  |  | LmiTr26269 | similar to CG8709-PA |
|  |  | LmiTr26411 | Tubulin beta chain |
|  |  | LmiTr27293 | Voltage-dependent calcium channel subunit alpha-2/delta-3 |
|  |  | LmiTr27965 | similar to protease, reverse transcriptase and RNase H |
|  |  | LmiTr28000 | glutathione-S-transferase-like protein |
|  |  | LmiTr28491 | RNA-directed DNA polymerase |
|  |  | LmiTr28568 | similar to CG31224-PA |
|  |  | LmiTr28853 | Serine-arginine protein |
|  |  | LmiTr28858 | Kinase suppressor of Ras 2 |
|  |  | LmiTr28934 | heterogeneous nuclear ribonucleoprotein k |
|  |  | LmiTr29122 | similar to ENSANGP00000024769 |
|  |  | LmiTr30 | similar to sallimus CG1915-PC |
|  |  | LmiTr32840 | calcium/calmodulin-dependent serine protein kinase membrane-associated guanylate kinase (cask) |
|  |  | LmiTr33265 | similar to Trafficking protein particle complex subunit 2-like protein |
|  |  | LmiTr33383 | diphosphomevalonate decarboxylase |
|  |  | LmiTr3355 | Dolichyldiphosphatase |
|  |  | LmiTr33628 | Enoyl-CoA hydratase domain-containing protein |
|  |  | LmiTr34831 | retinitis pigmentosa GTPase regulator-like protein |
|  |  | LmiTr351 | similar to predicted protein |
|  |  | LmiTr3510 | similar to Zinc finger protein 208 |
|  |  | LmiTr36224 | 7,8-dihydro-8-oxoguanine triphosphatase |
|  |  | LmiTr36934 | similar to GG1360 |
|  |  | LmiTr38126 | Lethal(3)malignant brain tumor-like 3 protein |
|  |  | LmiTr38320 | N-acetylgalactosamine kinase |
|  |  | LmiTr39791 | Serine/arginine repetitive matrix protein |
|  |  | LmiTr39940 | similar to Multidrug resistance protein homolog |
|  |  | LmiTr40891 | beta-1,3-glucanase |
|  |  | LmiTr4110 | Dedicator of cytokinesis protein 11 |
|  |  | LmiTr43405 | similar to ATP-binding cassette transporter |
|  |  | LmiTr44528 | heat shock protein 20.5 |
|  |  | LmiTr4480 | similar to pol-like protein |
|  |  | LmiTr45287 | neotenic-specific protein |
|  |  | LmiTr46042 | similar to UNC93A protein |
|  |  | LmiTr46410 | beta-glucosidase |
|  |  | LmiTr4746 | L-gulonolactone oxidase |
|  |  | LmiTr48926 | similar to GA18912-PA [Tribolium castaneum] |
|  |  | LmiTr49427 | similar to Peroxisomal biogenesis factor 6 |
|  |  | LmiTr49586 | mitochondrial import protein MMP37 |
|  |  | LmiTr4973 | reverse transcriptase-like |
|  |  | LmiTr50167 | VPS9-ankyrin repeat-containing protein |
|  |  | LmiTr50419 | similar to mediator complex |
|  |  | LmiTr51218 | Sodium-coupled monocarboxylate transporter |
|  |  | LmiTr51630 | similar to endonuclease-reverse transcriptase |
|  |  | LmiTr53345 | UDP-glucuronosyltransferase |
|  |  | LmiTr53529 | similar to GE16190 |
|  |  | LmiTr53712 | secretory Phospholipasennis] |
|  |  | LmiTr53747 | Zinc finger protein |
|  |  | LmiTr70136 | U6 snRNA-associated Sm-like protein |
|  |  | LmiTr70829 | dkey-189p24.5 proteinn adenosyltransferase-like |
|  |  | LmiTr71646 | Zinc carboxypeptidase |
|  | Stable Difference | LmiTr22731 | similar to WD repeat domain 24 |
|  |  | LmiTr52304 | similar to CG33556-PA |
|  |  | LmiTr26204 | similar to serine threonine-protein kinase |
|  |  | LmiTr37201 | Protein Malvolio （taste behavior） |
|  |  | LmiTr5765 | topoisomerase III alpha |
|  |  | LmiTr6611 | 26S proteasome regulatory subunit |
|  |  | LmiTr33504 | Multidrug resistance-associated protein 1 |
|  |  | LmiTr26373 | Protein Malvolio （taste behavior） |
|  |  | LmiTr9401 | protein arginine n-methyltransferase |
|  |  | LmiTr2211 | similar to CG11877-PA |
|  |  | LmiTr15663 | Annexin-B9 |
|  |  | LmiTr5089 | Conserved oligomeric Golgi complex subunit 4 |
|  |  | LmiTr6223 | nad dehydrogenase |
|  |  | LmiTr14894 | protein disulfide isomerase |
|  |  | LmiTr1326 | Conserved oligomeric Golgi complex subunit 4 |
|  |  | LmiTr3237 | proteasome 26S non-ATPase subunit 2 |
|  |  | LmiTr6169 | Transmembrane protein |
|  |  | LmiTr2139 | ubiquitin carboxyl-terminal hydrolase |
|  |  | LmiTr22245 | Staphylococcal nuclease (SNase-like), OB-fold |
|  |  | LmiTr18631 | Transcriptional adapter |
|  |  | LmiTr4499 | Exocyst complex component 4 |
|  |  | LmiTr13508 | Multidrug resistance-associated protein 1 |
|  |  | LmiTr17982 | DEAD box polypeptide 5 |
|  |  | LmiTr23897 | similar to lethal (3) 07882 CG5824-PA |
|  |  | LmiTr12249 | transposable element tc1 transposase |
|  |  | LmiTr20670 | 5'-AMP-activated protein kinase subunit beta-1 |
|  |  | LmiTr15378 | Fragile X mental retardation syndrome-related protein 1 |
|  |  | LmiTr43797 | Exocyst complex component 4 |
