# Supplementary material for: De Novo Analysis of Transcriptome Dynamics in the Migratory Locust during the Development of Phase Traits
Source: PLoS One. 2010 Dec 30;5(12):e15633. doi: 10.1371/journal.pone.0015633 (PMC3012706; doi:10.1371/journal.pone.0015633)
Supplement: Table S13 — Main signal pathways and events involved in the Group I of the major functional network. (DOC) [file pone.0015633.s027.doc]

**Table S13. Main signal pathways and events involved in the Group I of the major functional network**

| Pathways or Events | No. | CG no. of the *Drosophila* orthologue |
| --- | --- | --- |
| Signalling by NGF | 16 | CG5680, CG4201, CG5686, CG4006, CG6297, CG30440, CG3143, CG5092, CG7524, CG7397, CG14940, CG32498, CG9210, CG7793, CG10493, CG4141 |
| Signaling by GPCR | 13 | CG4006, CG4574, CG2835, CG30440, CG7397, CG11081, CG14940, CG32498, CG5411, CG9210, CG7793, CG17245, CG4141 |
| Axon guidance | 11 | CG5661, CG8874, CG4032, CG8355, CG6297, CG13521, CG7595, CG11081, CG7524, CG7793, CG17245 |
| Signaling by Rho GTPases | 9 | CG8948, CG4937, CG4755, CG8355, CG30440, CG7397, CG5410, CG1748, CG7793 |
| Integrin cell surface interactions | 7 | CG33196, CG1212, CG3936, CG7524, CG11326, CG1587, CG7793 |
| Signaling by Notch | 6 | CG33196, CG3936, CG6124, CG7147, CG10772, CG6127 |
