# Supplementary material for: De Novo Analysis of Transcriptome Dynamics in the Migratory Locust during the Development of Phase Traits
Source: PLoS One. 2010 Dec 30;5(12):e15633. doi: 10.1371/journal.pone.0015633 (PMC3012706; doi:10.1371/journal.pone.0015633)
Supplement: Table S14 — Differentially expressed genes between the two phases of 4th instar locusts in downstream events of GPCR signaling. Up and down means DETs (differentially expressed transcripts) up-regulated or down-regulated in the gregarious 4th instar locusts, respectively. (DOC) [file pone.0015633.s028.doc]

**Table S14.** **Differentially expressed genes between the two phases of 4th instar locusts in downstream events of GPCR signaling**

**Up and down means DETs (differentially expressed transcripts) up-regulated or down-regulated in the gregarious 4^th^ instar locusts, respectively.**

| Locust id | CG no. of the *Drosophila* orthologue | Name | Function in GPCR Signaling | DETs |
| --- | --- | --- | --- | --- |
| LmiTr6145 | CG11081 | plexA | protein binding; Transphosphorylation of pLIMK1 | up |
| LmiTr17444 | CG14940 | Pde1c | phosphodiesterase | up |
| LmiTr5224 | CG17245 | plexB | protein binding; Transphosphorylation of pLIMK1 | up |
| LmiTr2846 | CG2835 | G-salpha60A | G-salpha60A activate adenylate cyclase to produces cAMP and thus activate cAMP-dependent protein kinases | up |
| LmiTr19351 | CG30440 | CG30440 | guanyl-nucleotide exchange factor GEFs that activate Rho GTPase:GDP and RhoA,B,C | up |
| LmiTr15495 | CG32498 | dnc | Calmodulin activates Cam-PDE 1, cAMP hydrolysis | up |
| LmiTr3685 | CG4006 | Akt1 | Activated DAkt1 phosphorylates TSC2 which inhibits the TSC1/2 complex. AKT can phosphorylate forkhead box transcription factors, SRK, TSC2, CREB, eNOS, GSK3, IKKalpha | up |
| LmiTr19429 | CG4141 | Pi3K92E | PI3K produces PIP3 and other phosphatidyl inositides, PI3K catalytic subunit binds to Gab1:Grb2:PI3K:EGF:EGFR | up |
| LmiTr23528 | CG4574 | Plc21C | Activation of PLC beta-1/4, PIP2 hydrolysi | up |
| LmiTr5209 | CG5411 | Pde8 | cAMP degradation by Phosphodiesterases | up |
| LmiTr22648 | CG7397 | CG7397 | 554616 GEFs activate Rho GTPase:GDP, activate RhoA,B,C | up |
| LmiTr19399 | CG7793 | SoS | Sos Activation of Rac | up |
| LmiTr21514 | CG9210 | Ac13E | adenylate cyclase catalyses cAMP synthesis | up |
