# Supplementary material for: De Novo Analysis of Transcriptome Dynamics in the Migratory Locust during the Development of Phase Traits
Source: PLoS One. 2010 Dec 30;5(12):e15633. doi: 10.1371/journal.pone.0015633 (PMC3012706; doi:10.1371/journal.pone.0015633)
Supplement: Table S15 — Statistics of self-to-self similarity search results in different overlap ratios (identity >0.96). (DOC) [file pone.0015633.s029.doc]

**Table S15. Statistics of self-to-self similarity search results of different overlap ratios (identity >0.96).**

Total sequence number is 72,977.

| Overlap Ratio (%) | Cluster No. | Sequences Affected |
| --- | --- | --- |
| 60 | 56,204 | 16,773 |
| 70 | 59,164 | 13,813 |
| 80 | 62,226 | 10,751 |
| 90 | 65,973 | 7,004 |
| 95 | 68,552 | 4,425 |
