# Supplementary material for: De Novo Analysis of Transcriptome Dynamics in the Migratory Locust during the Development of Phase Traits
Source: PLoS One. 2010 Dec 30;5(12):e15633. doi: 10.1371/journal.pone.0015633 (PMC3012706; doi:10.1371/journal.pone.0015633)
Supplement: Table S16 — Primers used in the real time PCR experiment. (DOC) [file pone.0015633.s030.doc]

**Table S16. Primers used in the real time PCR experiment**

| Transcript Id | Sense (5’ to 3’) | Antisense (5’ to 3’) |
| --- | --- | --- |
| LmiTr5408 | CCGTCTATCGGAGGTAGCGT | CTCGGGTAGTTTATGTTCAGC |
| LmiTr18418 | CATTTGAGTACCTGGCCTATC | TTATCGAGCAATCCCGTCT |
| LmiTr16168 | GAGAGGTGGAAGACAATGGTT | GGCCTTTACTTTACTGCGTT |
| LmiTr10064 | ACCCAAACTACTTCCCGCATCC | ACATCAGCCCGAACCGCATT |
| LmiTr28183 | CGGACTGCTCTGACGGTT | TGTCTCAGGAGCACAGGTTG |
| LmiTr18955 | CGGCGGACAACGGCAAT | GACCAGACCCAGGGAGATG |
| LmiTr16741 | GAAGCAGCCATCAATGTAGC | GGGCAGCAGAGTTGTGAAG |
| LmiTr7275 | TGGGTGGCTGGTCTTTCG | CGCCTCACGCAGTTCTTTG |
| LmiTr10056 | CCGAGACCATCGCCAAAC | TCGCTGTCGTCCGTCATC |
| LmiTr17469 | GGAAAATGTGCCGAGTAGAGC | CGGTGCCAGGTTCGTAAGC |
| LmiTr15266 | TCGGAAAGCAACGTGAACTC | GACAGACGGTGGCGATAAAG |
| LmiTr6902 | TGGAGACGCCCCAGGAGAAC | GCCAGAGCCGGACCAGAAC |
